# Selected Abstracts from the Annual Meeting of SESAM - the Society for Simulation in Europe, 2025

**DOI:** 10.1186/s41077-025-00360-4

**Published:** 2025-06-24

## O1. A day at Coroner's court: Using Simulation to de-mystify the process for clinicians

### Format: Descriptive Work - Oral Presentations, Short Communications and E-posters

#### Topic: Addressing Emerging Healthcare Challenges

##### Cedar Andress

###### Southern Health and Social Care Trust (Hospital in Craigavon, Northern Ireland)

*Advances in Simulation 2025,* **10(1)**:01

**Introduction Setting, background and identification of needs leading to the initiative**

Coroners are judges who investigate the cause of deaths reported to them. Inquests can be called for several reasons, including when a death is sudden, unexplained or potentially suspicious. The purpose is to identify the cause of death, as well as the circumstances surrounding it, including whether the death was preventable.

Unfortunately, a significant number of us as healthcare professionals will be involved in a coroner’s inquest during our professional careers. Understandably, this process can often cause significant stress and worry for a protracted time. Most staff have little to no comprehension of the process, nor experience of reporting writing or presenting oral evidence in court and are not provided with formal training during their degree.

**Description of initiative and approach/methods used**

I am creating a course with the objective of de-mystifying the inquest process and increasing psychological safety, providing a safe environment for doctors to learn and ask questions. This day will include information on the inquest process and roles of the professionals, advice on report writing and presenting oral evidence, the impact on families and doctors, where to access support and a Simulated coroner's court and debrief.

A scoping exercise and informal interviews were carried out to understand doctor's apprehension around coroner's court and direct the designing of the day including identifying set Learning Objectives. The Scottish Centre debrief model was used to design and debrief the simulation. The scenario was written with help from the medical advisor to the coroner and the health board's legal team.

**Discussion of the impact/outcome, and novelty of the initiative**

Course has not yet been held - for April/May 2025, A pilot may be held prior to the day to iron out any issues.

This appears to be the first course of its kind in Northern Ireland and the first course using simulation for coroner's court learning, perhaps in the UK?

**Keywords**

Coroner, Inquest, Legal, Simulation

**References/Acknowledgements**

- Madireddy S, Rufa EP. Maintaining confidentiality and psychological safety in medical simulation. StatPearls [Internet]. 2023 May 1.
- Lateef F. Maximizing learning and creativity: understanding psychological safety in simulation-based learning. Journalof emergencies, trauma, and shock. 2020 Jan 1;13(1):5–14.
- Beggs R, McKay I, Linsley P. The development of simulated learning environments involving coroner’s courtattendance in mental health nursing education. Mental Health Practice. 2024 May 2;27(3).

## O2. An Innovative National Strategy: Sim-based Mastery Learning for Acquisition of Emergency Medicine HALO Procedural Skills

### Format: Descriptive Work - Oral Presentations, Short Communications and E-posters

#### Topic: Curriculum Development and Assessment

##### Laura McGregor^1^, Josh Shaw^2^, Kate Mitchell^3^, Ben Clarke^3^

###### ^1^NHS Lanarkshire (Scotland), NHS Education for Scotland; ^2^NHS Forth Valley (Scotland, Larbert); ^3^NHS Lothian (Scotland)

*Advances in Simulation 2025,* **10(1)**:02

**Introduction: setting, background and identification of needs leading to the initiative**

Emergency Medicine (EM) physicians must be able to perform emergency skills which are often time-critical and can be life-saving, as stipulated in SLO6 of the RCEM Higher Specialty Training (HST) curriculum(1). There are significant challenges in ensuring these skills are taught reliably in practice due to the High Acuity Low Occurrence (HALO) of many of these events.

We designed, developed and delivered an innovative Scotland-wide strategy using modified simulation-based mastery learning (SBML) methodology for HALO procedures as a proposed solution to this issue.

**Description of initiative and approach/methods used**

As part of Scotland's simulation strategy for EM HST, we have produced five training days which we envisage all trainees will attend through their training. Each has an RCEM curriculum-linked focus: trauma & orthopaedics (T&O), resuscitative thoracotomy, head and neck procedures, cardiorespiratory procedures and obstetric & neonatal emergencies. Along with other teaching methodologies (workshops, skills and drills, immersive simulation), the days adopt SBML to enable trainees to evidence acquisition of skills and competencies in common and HALO procedures. Some procedures, for example intercostal drain insertion, lend themselves well to SBML method used widely in the national Internal Medical Training programme (2). For procedures such as resuscitative hysterotomy, thoracotomy and facial trauma management, we have modified the SBML process to account for the HALO nature of these events and created new Mastery pre-learning videos and reading packs. The main alteration is the overall goal: our method aims to have the trainee feel equipped to perform the procedure in real life on their next shift; rather than “ready for supervised practice”.

Furthermore, for facial trauma and thoracotomy, our innovative strategy incorporates cadaveric models into the modified SBML process with the aim of enhancing fidelity.

**Discussion of the impact/outcome, and novelty of the initiative**

We have completed successful pilot courses in four out of five of the aforementioned topics: T&O; cardiorespiratory; obstetric & neonatal and resuscitative thoracotomy.

The standard SBML model was adopted for large joint fluid aspiration, open and Seldinger intercostal drain insertion.

Our innovative modified SBML model for HALOs was applied to resuscitative hysterotomy and thoracotomy; the latter making use of cadaver models.

Feedback gathered from these pilot courses was overwhelmingly positive, on average resulting in a shift from pre-course confidence of 2.5 to 4.5 post-course This equates to RCEM entrustment level of 3 (“supervisor ‘on call’ from home for queries” (1)).

These exciting successful pilot courses suggest SBML can be used to enhance skill acquisition for EM HSTs. The innovation of our modified SBML method, along with use of cadaver models, may be of particular value in HALO procedures.

**Keywords**

Sim-based Mastery Learning, Emergency Medicine, Training, HALO

**References/Acknowledgements**

- 1. Royal College of Emergency Medicine (2019). Programme of Assessment. [online] RCEMCurriculum. Available at: https://rcemcurriculum.co.uk/assessment/
- 2. McAleer P, Tallentire VR, Stirling SA, Edgar S, Tiernan J. Postgraduate medical procedural skills: attainment of curricular competencies using enhanced simulation-based mastery learning at a novel national boot camp. Clinical Medicine. 2022 Mar;22(2):125–30.

## O3. Development and validation of a distance- based training program of Basic Cardiac Life support for non-medical personnel and comparison with traditional in person methodology: A non-inferiority study

### Format: Research Studies - Oral Presentations, Short Communications and E-posters

#### Topic: Addressing Emerging Healthcare Challenges

##### Marcia Corvett, David Acuña, Jerónimo Rojas, Andrés Schneider, Julian Varas, Sofia Abedrapo, Elga Zamorano, Carlos Obreque, Cristián Jarry, Fernando Altermatt

###### Pontificia Universidad Católica (Santiago, Chile)

*Advances in Simulation 2025,* **10(1)**:03

**Introduction: context and hypothesis/aims**

This study aimed to compare a distance-based training program with asynchronous feedback about Cardiopulmonary Resuscitation (CPR) for non-medical personnel with traditional Basic life support (BLS) in person course. The hypothesis is that distance-based program is non inferior to the traditional one, maintaining competitive costs.

**Methods and results: description of the methods used/study design/data collection. Presentation of the results addressing the study hypothesis/aims**

**Methods**

After approval by the ethics committee, 192 non-medical personnel were recruited to participate in this protocol.

Participants were randomized to 2 types of training:

- Traditional BLS in person course of the American Heart Association (T-course).
- Distance-based course with asynchronous distance feedback through an online platform (D-course).

For the distance-based course, materials for practice were mailed to participants home address (pad, resuscitation torso, an automated external defibrillator). A video-based remote asynchronous feedback platform was used. The course was structured in stages in the platform, with theoretical and practical steps. Participants reviewed instructional videos, practiced unsupervised, uploaded their practice videos, and received asynchronous feedback from instructors.

This cycle continued until they met the approval criteria.

Both groups underwent a pre-training evaluation (PRE) and a post-training evaluation (POST). For both assessments participants were videotaped during a CPR. Videos were evaluated by two independent and blinded reviewers, who rated participant’s performance using the AHA CPR and AED Skills Testing Checklist. Additionally, the quality of chest compressions (CC) was assessed using the Laerdal simulator application.

A sample size of 118 subjects was calculated to detect a non-inferiority margin of 2 points of the checklist, with a significance 0.05 and power of 0.8. Data are expressed in means and SD. A paired t-test was used to find differences.

**Results**

One hundred twenty-four participants completed the training and the assessments.

Demographics shown no differences between groups.

Mean checklist scores improve from 3 (2) points to 9 (3) in T-course and from 3 (2) to 10 (3) in D-course. There were no significant differences in POST assessment between courses.

Mean CC rate improve from 94 (33)/min to 113 (13.5)/min in T-course and from 90 (55)/min to 108 (13.8)/min in the D-course. Mean CC depth increase from 31 (2.9) mm to 58 (3.4) mm in T-course and from 29 (3) mm to 59 (2.8) mm in the D-course.

Individual costs were 68.56 for the T-course versus 70.67 for the D-course in USD.

**Discussion of the impact/outcome, and novelty of the Research**

Both training programs improves significantly participants’ proficiency in CPR. The distance-based course with asynchronous feedback through C1DO1 platform was non inferior to the traditional BLS course, maintaining competitive costs.

**Keywords**

Resuscitation, Cardiopulmonary resuscitation, CPR, Chest compression, Basic life support, Simulation; Distance-based simulation, Simulation-based training,

**References/Acknowledgements**

- 1.- Vera M, Kattan E, Cerda T, Niklitshek J, Montaña R, Varas J, Corvetto MA. Implementation of Distance-Based Simulation Training Programs for Healthcare Professionals: Breaking Barriers During COVID-19 Pandemic. Simul Healthc. 2021 Dec 1;16(6):401–406. 10.1097/SIH.0000000000000550.
- 2.- Corvetto MA, Kattan E, Varas J, Caro I, Altermatt FR. Designing Sustainable Solutions to Implement a Distance-Based Simulation Basic Life Support Training Program During COVID-19 Pandemic in Low-Income Countries. Simul Healthc. 2022 Mar 8. 10.1097/SIH.0000000000000651.

## O4. Estudio de fiabilidad y validez del instrumento NEUMOBACT para valorar el desempeño de prevención de infecciones de las enfermeras de cuidados intensivos en escenarios de simulación

### Format: Research Studies - Oral Presentations, Short Communications and E-posters

#### Topic: Patient Safety and Quality Improvement

##### Mariona Farres-Tarafa^1^, Marta Raurell-Torredà^1^, Ignacio Zaragoza-García^2^, Oscar Arrogante^2^, Anna María Aliberch-Raurell^3^, Francisco Javier Sánchez-Chillón^4^, Martín Torralba-Melero^5^, Andrés Rojo-Rojo^6^

###### ^1^Universitat de Barcelona (Barcelona,Spain); ^2^Universidad Complutense de Madrid, (Madrid, Spain); ^3^Hospital Clínic de Barcelona (Barcelona, Spain); ^4^Hospital Universitario 12 de Octubre (Madrid, Spain); ^5^Hospital General Universitario de Albacete (Albacete, Spain); ^6^niversidad Católica de Murcia, Campus de los Jerónimos (Guadalupe, Murcia, Spain)

*Advances in Simulation 2025,* **10(1)**:04

**Introduction: context and hypothesis/aims**

A raíz de la pandemia por el virus SARS-CoV-2, las tasas de incidencia de neumonía asociada a la ventilación mecánica (NVM) y bacteriemia relacionada con catéter (BRC) se incrementaron entre dos y tres veces. Entre otras medidas, el consejo de expertos del Ministerio de Sanidad propuso “promover la formación del personal sanitario de las UCI”.

Des del grupo de trabajo de Simulación de Sociedad Española Enfermería Intensiva Unidades Coronarias (SEEIUC) se diseñó un curso formativo, denominado SIMULAZERO basado en simulación formato Evaluación Clínica Objetiva Estructurada (ECOE) para comprobar la traslación de la teoría a la práctica clínica de los conocimientos y habilidades relacionados con prevención NVM y BRC (1). Dentro de este curso se creó un checklist, llamado NEUMOBACT. El objetivo del estudio fue analizar la fiabilidad interobservador del checklist NEUMOBACT para comprobar si reproduce resultados consistentes cuando se aplica en diferentes ocasiones.

**Methods and results: description of the methods used/study design/data collection. Presentation of the results addressing the study hypothesis/aims**

Estudio descriptivo observacional de carácter métrico, que ha seguido las indicaciones de GRRAS (checklist for reporting of studies of reliability and agreement) (2). La concordancia interobservador se analizó mediante el Coeficiente Kappa de Gwet para cada ítem del checklist NEMOBACT y también para el total de cada estación.

Se recogieron 95 pares de checklist NEUMOBACT válidos. En la estación Inserción de Catéter Venoso Central, Kappa de Gwet para el total fue de 0.934 (CI 95% [0.919–0.949]). Solo 2 de los 17 ítems puntuaron inferior a 0.9. En la estación de aspiración de secreciones fue de 0.869 (CI 95% [0.851 -– 0.886]). De los 26 ítems que conformaban esta estación, 16 mostraron un porcentaje de acuerdo superior a 0.9. En la estación Cuidados del Paciente, fue de 0.911 (CI 95% [0.896 - 0.927]). De los 21 ítems, 17 mostraron un porcentaje de acuerdo superior a 0.9 y 4 entre 0.810 y 0.894.

**Discussion of the impact/outcome, and novelty of the Research**

El checklist NEUMOBACT puede ser útil para evaluar las habilidades antes y después de la formación en medidas de prevención de NVM y BRC y para reforzar durante el debriefing, la evidencia científica en la que se basan las decisiones de aquellos ítems que se han realizado incorrectamente.

La fiabilidad interobservador del checklist NEUMOBACT indica un acuerdo sustancial entre parejas de observadores, siendo validada en una muestra amplia de enfermeras de UCI.

**Keywords**

Catheter-related infections, checklist, clinical skills, critical care nursing, cross infection, high-fidelity simulation training, nursing education, patient safety, validation study, ventilator- associated pneumonia

**References/Acknowledgements**

1. Raurell-Torredà M, Zaragoza-García I, Aliberch-Raurell AM, Sánchez-Chillón J, Torralba-Melero M, Arrogante O, etal. SIMULAZERO: taller de simulación para actualizar conocimientos y habilidades en la prevención de la neumonía asociada a ventilación mecánica y bacteriemia relacionada con catéter (Proyectos Zero). Enferm Intensiva. septiembre de 2022;33:S45-55.
2. Kuiper JH. GRRAS checklist for reporting of studies of reliability and agreement.

**Fig. 1 (O4):**
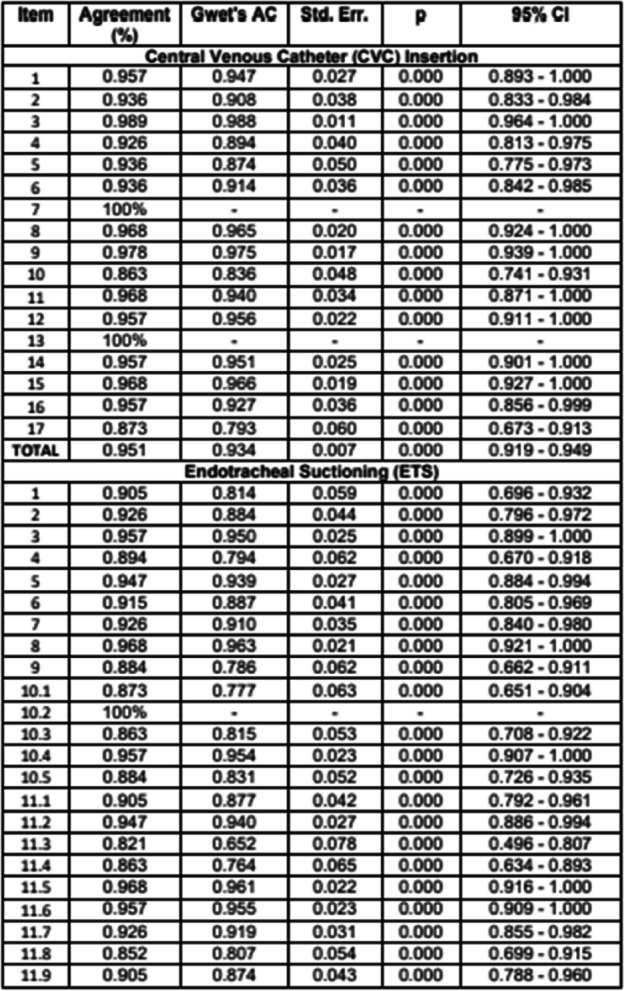
See text for description

**Fig. 2 (O4):**
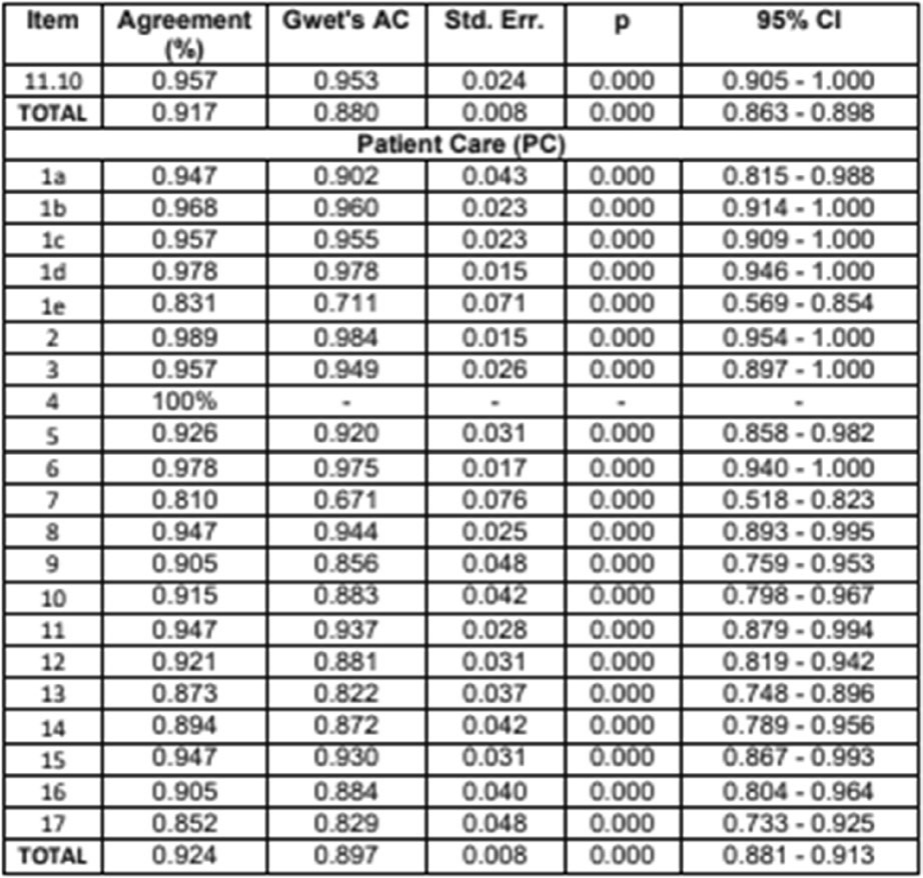
See text for description

## O5. From Macbeth to Magills: enhancing learner psychological safety and wellbeing in undergraduate Paramedic simulation through applied drama and theatre

### Format: Descriptive Work - Oral Presentations, Short Communications and E-posters

#### Topic: Culture, Wellbeing, Equity, Diversity, Inclusivity

##### Katie Pavoni, Caroline Neveu

###### City St George's University of London (Northampton Square, London)

*Advances in Simulation 2025,* **10(1)**:05

**Introduction: setting, background and identification of needs leading to the initiative**

Tiered simulation is a core pedagogical approach within our BSc Paramedic Science. Fostering a psychologically safe learning environment is essential to enable learners to promote wellbeing and overall transformative learning (1). Despite substantial investment from faculty in this domain, however it was recognised that learners can often still feel a heightened sense fear and self-consciousness when undertaking simulation particularly when commencing higher education (HE).

HE is associated with a significant period of transition, with learners being distanced from support networks, forming new social relationships and forming personal identity (2). This combined with the challenging nature of Paramedicine, can lead to increased vulnerability during simulation. Recognition of this complexity and adapting psychological safety approaches is essential to ensure preparedness for learning and practice.

**Description of initiative and approach/methods used**

This initiative saw 100 newly enrolled first year paramedic undergraduate learners and faculty undertake a day of facilitated drama and theatre activities led by the team of professional actors and simulated patients who work within the BSc Paramedic Science. This session was embedded within the induction curriculum of the first week of study, prior to the first exposure to peer learning and practical clinical simulation. Learners were asked to informally and anonymously consider their emotional state regarding simulation using a Scherer's Circumplex model (3).

Scaffolded applied drama activities were adopted, which included ice-breakers, movement and physicality, trust and communication games, debating activities and an improvisation session.

**Discussion of the impact/outcome, and novelty of the initiative**

Learners were actively encouraged to experiment, improvise, take risks and make mistakes in a light-hearted environment, free from judgement and supported by trust and mutual positive regard. To challenge the perceived hierarchy between academic faculty and learners and to encourage authentic participation it was essential that paramedic educators also took part in activities alongside learners. Through this role-modelling of vulnerability, inclusivity and suspension of inhibition, learners were able to foster new peer connections, minimise apprehension of working with simulated patients and actively shape the culture of their future learning environment and community.

These elements are central to ensuring psychological safety within simulation and this approach also allowed for the prelude for key concepts such as introduction of the ‘fiction contract’ and ‘basic assumption’ as discussed by Rudolph et al (2014) (4)

Students described a subsequent shift to positive emotional states, which invite openness to learning (5) and a sense of curiosity and willingness to partake in curriculum simulation.

These innovative sessions help launch a positive learning culture, communication and personal resilience, fundamental aspects of Paramedicine.

**Keywords**

Resilience, Psychological Safety, Wellbeing, Culture

**References/Acknowledgements**

1. Purdy, E., Borchert, L., El-Bitar, A. et al. Taking simulation out of its “safe container”—exploring the bidirectionalimpacts of psychological safety and simulation in an emergency department. Adv Simul 2022, 7, 5 (2022) 10.1186/s41077-022-00201-8
2. McLafferty M, Lapsley CR, Ennis E, et al. Mental health, behavioural problems and treatment seeking among studentscommencing university in Northern Ireland. PLoS One. 2017;12(12). 10.1371/journal.pone.0188785
3. Scherer, K.R, What are emotions? And how can they be measured? Social Science Information, 2005; 44, 695—729.
4. Rudolph JW, Raemer DB, Simon R. Establishing a safe container for learning in simulation: the role of the presimulation briefing. Simul Healthc. 2014;9(6):339–349. 10.1097/SIH.0000000000000047

5. Perrmann-Graham J., Liu J., Spataro SE., Fostering psychological safety: using improvisation as a team building tool in management education, International Journal of Management Education, 2022; 20(2). 10.1016/j.ijme.2022.100617

## O6. Pass the bleep: Using simulation-based training in preparation for healthcare industrial action

### Format: Descriptive Work - Oral Presentations, Short Communications and E-posters

#### Topic: Interprofessional/Team Education and Training

##### Kathryn Mullan, Carol McCBenjamin McNaughten, Clare Loughran, Peter Mallett, Andrew Thompson, Thomas Bourke

###### SimEd team, Royal Belfast Hospital for Sick Child ren (Belfast, Northern Ireland)

*Advances in Simulation 2025,* **10(1)**:06

**Introduction: setting, background and identification of needs leading to the initiative**

This year, junior doctors in Northern Ireland seek to secure pay restoration in what has been described as the ‘most disruptive industrial action in NHS history’ [1]. In response to this, consultant teams across the province have stepped up to cover on-call rotas, ensuring the safety of patients and the delivery of essential services during this period. To strengthen these efforts, junior doctors, along with advanced nurse practitioners, ran a series of simulation-based training sessions for consultant teams. These sessions aim to reinforce local protocols, revisit practical skills, and refresh paediatric emergency training in preparation for on-call duties.

**Description of initiative and approach/methods used**

We aimed to assess if simulation-based interprofessional education is an effective training tool for systems learning in an effort to improve consultant team ‘readiness’ for industrial action.

Over a three-day period, 18 senior staff members (17 consultants and 1 SAS doctor) participated in an 80-minute simulation training session. The simulation sessions used locally developed scenarios to apply local practices and APLS principles. Equipment included high-fidelity mannequins, basic airway devices, IV access, monitoring and a defibrillator in the setting of a highly immersive simulation suite. A mixed-methods questionnaire was distributed to participants before and after the simulation training session, assessing consultants’ confidence levels, knowledge, and attitudes towards the impending junior doctor strikes.

**Discussion of the impact/outcome, and novelty of the initiative**

Three main themes, each with several subgroups, were extracted and identified from the free text responses. The themes highlighted the perceived benefits of using simulation in preparation for industrial action, including the improved recognition of local protocols (rapid identification of contact details for on-call teams, referral pathways and local guidelines), re-familiarisation with practical skills and equipment (defibrillator, cannulation and administration of emergency drugs) and improvement in human factor skills among newly-formed consultant teams (leadership, communication, resource utilisation, workload distribution, situational awareness, and management of disruptions). Staff involved found this simple, convenient, and low-cost interprofessional simulation training instrumental in improving their sense of ‘readiness’ for industrial action by addressing the logistical challenges of managing paediatric care in the absence of junior staff.

Our simulation-based training intervention was well received by consultant teams in preparation for the challenges of service delivery during junior doctor strikes. Satisfaction was high, and the simulation exercise increased their confidence by revising practical skills and revisiting protocols commonly utilised by junior staff. With further industrial action on the horizon, we aim to roll out our simulation-based training programme to consultant teams on a larger scale.

**Keywords**

N/A

**References/Acknowledgements**

- Financial Times. Junior doctors’ strike will be ‘most disruptive in NHS history’, Health Leaders Warn. Available at: https://www.ft.com/content/59884863-d504-48ce-9974-8ccc296823f7 (Accessed: 09 May 2024).

**Fig. 1 (O6):**
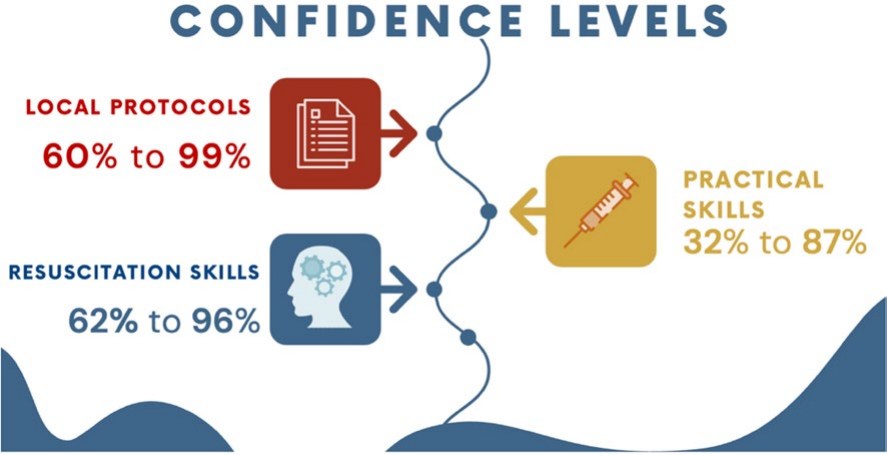
See text for description

## O7. ‘No such thing as innocent bystanding’ (1): immersive active bystander (iABT) training for medical students

### Format: Descriptive Work - Oral Presentations, Short Communications and E-posters

#### Topic: Culture, Wellbeing, Equity, Diversity, Inclusivity

##### Stephen Harte, Linda Ni Chianain, Catherine Murphy, Aoife Rafferty, Paul Murphy, Gerry Gormley

###### Queen's University (Belfast. Belfast, Northern Ireland)

*Advances in Simulation 2025,* **10(1)**:07

**Introduction: setting, background and identification of needs leading to the initiative**

If left unchallenged, poor behaviours can negatively impact healthcare workers (HCWs) and their ability to provide quality healthcare. It is fundamental that HCWs create a respectful workplace and address issues such as discrimination. Active bystander training (ABT) aims to empower staff to challenge inappropriate behaviours. Often delivered online or via roleplay, ABT helps individuals understand the principles of intervening. However, situations involving inappropriate behaviour can be highly emotive and challenging to intervene. Developing tacit knowledge (i.e., innate knowledge) is crucial for effectively applying the principles of ABT. Simulation provides guided experiential learning to enhance cognitive, emotional, and behavioural skills. Our teaching innovation, leverages immersive training by drawing on the principles of Forum Theatre (FT) to deliver immersive ABT (iABT).

**Description of initiative and approach/methods used**

A team of academics/medical students designed a FT play [2]. Drawing on real-life experiences, we created a play depicting racist comments directed to a junior doctor from an underrepresented ethnic group. The session began with an overview of ABT and heuristic tools (e.g., the 4 Ds of intervening). This was followed by the FT performance with SPs. Starting with an ‘anti-model’ scene, students were introduced to the characters and situation. In the next act, students observed inappropriate behaviour from a patient (the antagonist) towards the junior doctor (the protagonist) and reactions of the bystander portrayed as ‘medical students’. Following a group discussion, the play was re-enacted, with, students (the audience) taking control as"Spect-actors."They could intervene at any point by ‘clapping’, either to step into the scene themselves or direct the SPs to take a different course of action. A ‘joker’ facilitated the FT, guiding the interactions. After the re-enactment, the session concluded with a group debrief.

**Discussion of the impact/outcome, and novelty of the initiative**

Evaluatory feedback has been overwhelmingly positive. To date 136/304 students have participated (full results will be presented at meeting). On a scale of 1 (poor) - 5 (excellent): the session received an overall rating of 4.7/5. In terms of the perceived ‘importance’ 4.8/5, and engagement 4.8/5. Additionally, 4.6/5 felt more empowered to actively intervene. Free-text feedback reinforced these positive quantitative evaluations. Students found the session highly interactive, immersive, and safe learning space. They reported gaining a deeper understanding on how to ‘step forward,’ and the various ways of intervening, and feeling compelled ‘to do something.’ As poet laureate Seamus Heaney said, “There is no such thing as innocent bystanding [1],” and our iABT seems to have enhance medical students'confidence and desire to intervene.

**Keywords**

Simulation, active bystanding, Forum Theatre

**References/Acknowledgements**

1. Mycenae lookout (Cassandra) (The Spirit level 1996) Seamus Heaney 2) Boal, Augusto."Games for actors and non-actors."(1992).

## O8. A Lived Experience Partnership in Simulation-based Continuing Professional Development: A case study for ethical co-production

### Format: Descriptive Work - Oral Presentations, Short Communications and E-posters

#### Topic: Culture, Wellbeing, Equity, Diversity, Inclusivity

##### Stephanie Sliekers, Fabienne Hargreaves, Faith Rockburne, Howard Fruitman, Rachel Antinucci, Petal Abdool.

###### Centre for Addiction and Mental Health (London, England)

*Advances in Simulation 2025,* **10(1)**:08

**Introduction: setting, background and identification of needs leading to the initiative**

People with Lived Experience (PWLE) are those who have been impacted by mental illness and/or addiction, and share their lived-expertise to create and enhance healthcare educational programs. Ethical co-production in medical education for healthcare professionals and trainees is a way to bridge the gaps in perspective and imbalances of power between patients and medical professionals in the healthcare system [1]. Co-production in all stages of simulation development not only ensures accurate patient portrayals, it can also integrate a variety of medical and psychiatric perspectives, and augments the focus of trauma-informed, patient-centered practices in simulation and psychiatric medicine more broadly [1, 2]. While ethical collaboration with PWLE is imperative in simulation, there is fear and uncertainty of engaging with PWLE, and balancing inherent power differences [3].

**Description of initiative and approach/methods used**

The Centre for Addiction and Mental Health Simulation Centre, in collaboration with PWLE, have co-developed a successful working model emphasizing co-production throughout all stages of simulation development, implementation and evaluation. This presentation will share findings from an evaluation approach, guided by Moore’s Outcome Evaluation Framework, of the ethical collaboration process in simulation, and share co-production frameworks, best practices and guidelines [4].

**Discussion of the impact/outcome, and novelty of the initiative**

We surveyed simulation faculty about their experience collaborating with PWLE in the development and delivery of simulation-based education. All faculty (100%) surveyed were satisfied/very satisfied with their experience working with PWLE and would recommend PWLE be involved in future projects (n=19). Faculty reported the co-production process enhanced empathy and compassion when communicating with patients and staff, provided them with new insights into the patient perspective, and enhanced the realism of both the simulated patient portrayals and scenarios. Over 90% of learners agreed/strongly agreed that participating in simulations that included PWLE provided them with new skills, enhanced the need for compassion when communicating with patients, and provided them with new insights into the patient perspective (n=52). Our evaluation found that collaborating with PWLE in the development and delivery of simulation-based education provided valuable learning for both faculty and learners. Ethical co-production, however, requires a significant investment of time, resources, funding, staff, and relationship-building that can take many years to formally establish. We have found that starting with a clear framework for co-production, developing infrastructure to support that framework, hiring and onboarding PWLE, determining ethical co-production practices, and performance and professional development have been key enablers to overcome barriers to ethical co-production. This model illustrates a collaboration with PWLE that other healthcare professional education programs can learn from and replicate.

**Keywords**

Mental Health

**References/Acknowledgements**

1. Kneebone R, Weldon SM, Bello F. Engaging patients and clinicians through simulation: rebalancing the dynamics ofcare. Adv Simul (Lond). 2016;1:19. Published 2016 Jun 15. 10.1186/s41077-016-0019-9.
2. Jha V, Quinton ND, Bekker HL, Roberts TE. Strategies and interventions for the involvement of real patients inmedical education: a systematic review. Med Educ. 2009;43(1):10–20. 10.1111/j.1365-2923.2008.03244.x.
3. Happell B, Byrne L, McAllister M, et al. Consumer involvement in the tertiary-level education of mental health professionals: a systematic review. Int J Ment Health Nurs. 2014;23(1):3–16. 10.1111/inm.12021.

## O9. Aplicación de modelos de inteligencia artificial para mejorar la competencia de alumnos de medicina en la redacción de historias clínicas tras escenarios de simulación

### Format: Research Studies - Oral Presentations, Short Communications and E-posters

#### Topic: Research Methodolgy

##### Emilia Cervera Barba, Sophia Denizon Arranz, Alonso Mateos Rodriguez, Ana M Maitin Lopez, Alberto Nogales Moyano, Enrique Aranguren, Alvaro J Garcia Tejedor

###### Universidad Francisco de Vitoria (Madrid, Spain)

*Advances in Simulation 2025,* **10(1)**:09

**Introducton: context and hypothesis/aims**

Los alumnos de Medicina deben adquirir competencia en la redacción de historias clínicas (HC), por su importancia legal y profesional. El entrenamiento con pacientes simulados mejora su capacitación clínica y adicionalmente les da oportunidad de redactarlas. Una dificultad de los profesores de simulación es la falta de tiempo para corregir todas las HC, posteriormente al debriefing. El uso de modelos de inteligencia artificial (IA) podría suponer una ayuda para la corrección y, consecuentemente, mejorar la capacitación de los alumnos al proveerles de un feedback individualizado cada vez que redacten una HC.

**Methods and results: description of the methods used/study design/data collection. Presentation of the results addressing the study hypothesis/aims**

Se entrenaron varios Large Language Models (LLM, LLaMa-2-13B y 7B, GPT-3.5 y GPT-4.0) con 47 textos de semiología médica y 50 HC redactadas por alumnos y corregidas por profesores de simulación clínica en base a una rúbrica de 48 ítems. Los modelos se entrenaron con los documentos en español o traducidos al inglés, según necesidad. Inicialmente, la máxima concordancia en la corrección realizada por un LLM respecto a los profesores fue del 83,56 ± 7,11% de los ítems para GPT-4.0. Se incluyeron técnicas de Fine-Tunning y LoRA para adaptar LLaMa-2-7B a la tarea de corrección de HC, alcanzando una concordancia de corrección del 72,50 ± 8,11% de los ítems. Según las distintas técnicas, se consiguió que 28 de los 48 ítems tuvieran una tasa de error de interpretación baja (0–25% de las HC). Los errores más frecuentes de interpretación se produjeron en los ítems donde la ausencia de alergias, cirugías o intolerancias alimentarias era malinterpretada como omisión de recogida de información por el alumno.

**Discussion of the impact/outcome, and novelty of the Research**

En este trabajo se probaron distintos LLM y técnicas de reajuste para el objetivo de encontrar una herramienta válida de ayuda para el profesor y feedback para el alumno en la redacción de sus HC, consiguiendo unos resultados que demuestran que es una opción muy prometedora. La opción de trabajar con LLaMa-2, de acceso gratuito frente a GPT-4, la hace especialmente atractiva al lograr, con los sucesivos reentrenamientos, unas concordancias de corrección aceptables. Actualmente estamos reentrenando y reajustando las metodologías con las nuevas versiones de

LLM, cada vez más perfeccionados. En una fase posterior hemos incluido comentarios de feedback en los ítems de la HC para que los LLM puedan elaborar informes de evaluación formativa para los alumnos.

**Keywords**

Inteligencia artificial, historias clinicas, grado

**References/Acknowledgements**

none

## O10. Apologizing with H.E.A.R.T®: An innovative simulation to increase confidence in Disclosing a Patient Safety Incident

### Format: Descriptive Work - Oral Presentations, Short Communications and E-posters

#### Topic: Patient Safety and Quality Improvement

##### Petal Abdool, Julia Duzdevic, Stephanie Sliekers, Fabienne Hargreaves, Faith Rockburne, Alexandra Andric, Rachel Antinucci, Jennifer Grinfeld, Stephen Lincoln

###### Centre for Addiction and Mental Health (London, England)

*Advances in Simulation 2025,* **10(1)**:010

**Introduction: setting, background and identification of needs leading to the initiative**

Effective disclosure discussions of critical incidents are a hallmark of patient-centered care and safety [1]. Hospital staff must demonstrate accountability when safety incidents transpire and take necessary steps to mitigate re-occurrence [1]. The Canadian Disclosure Guidelines recommend staff receive ongoing education, mentoring and coaching to help facilitate effective disclosure discussions [1]. A Simulation training was developed to allow staff the opportunity to practice making an apology utilizing the H.E.A.R.T® communication model and discussing safety incidents with patients/clients and family members in a safe, supportive environment. This work is tied to our hospital’s Quality Improvement Mission, demonstrating organization commitment to support our disclosure of safety events.

**Description of initiative and approach/methods used**

The learning objectives of the training are to: describe the Quality and Adverse Event Process requiring a disclosure, develop a plan for a disclosure conversation, and communicate a disclosure to a substitute decision maker using the H.E.A.R.T.® model. Using the evidence based H.E.A.R.T.® communication model [2], staff compose a script for disclosure and practice it with a simulated participant (SP) who plays the role of a family member. Engaging with the SP helps staff learn how to foster a collaborative environment where all parties involved feel heard and their contributions are valued. This simulation training combines didactic teaching, safe setting with a simulated family member coupled with direct observation by a facilitator and lived experience advisor and then a debrief. The simulation uses a Rapid Cycle Deliberate Practice Approach [3]. Data was collected through self-reported pre-and-post training surveys. These surveys are guided by Moore’s Outcome Evaluation Framework, gathering data at multiple outcome levels including level 1 (participation), level 2 (satisfaction), and level 3 (declarative/procedural knowledge) [4].

**Discussion of the impact/outcome, and novelty of the initiative**

Nineteen healthcare providers have participated in the training. Data was collected from 14 clinicians between March-September 2024. Changes in confidence were measured across a 5-point Likert scale. The percentage of change was calculated using the mean pre-score (M=2.4, SD=0.99) and mean post-score (M=3.9, SD=0.77) across the 5 learning objectives, which was 60% for this group of learners. All participants (100%) reported intention to change practice after the training. Finally, all participants (100%) thought the inclusion of lived experience advisors provided new insight into the patient perspective and enhanced the need for compassion when communicating with patients and families. Evaluation results highlight the value of this simulation on learner’s confidence in disclosing a safety event. The inclusion of lived experience advisors in the debrief provided a unique opportunity for staff to learn from different perspectives.

**Keywords**

Mental Health

**References/Acknowledgements**

- 1. Disclosure Working Group. Canadian disclosure guidelines: being open and honest with patients and families. Edmonton, AB: Canadian Patient Safety Institute; 2011.
- 2. Cleveland Clinic. Communicate with H.E.A.R.T. 2024. Accessed February 25, 2024. https://my.clevelandclinic.org/departments/patient-experience/depts/experience-partners/licensed-programs/communicate-with-heart
- 3. Peng CR, Schertzer K. Rapid Cycle Deliberate Practice in Medical Simulation. In: StatPearls. Treasure Island (FL): StatPearls Publishing; July 24, 2023.
- 4. Moore DE Jr, Green JS, Gallis HA. Achieving desired results and improved outcomes: integrating planning andassessment throughout learning activities. J Contin Educ Health Prof. 2009;29(1):1–15.

## O11. Describing the use of simulation to evaluate undergraduate nursing student competency: Results of an international survey

### Format: Research Studies - Oral Presentations, Short Communications and E-posters

#### Topic: Curriculum Development and Assessment

##### Marian Luctkar-Flude^1^, Beth Rogers^2^, Jane Tyerman^3^, Suzanne Hetzel Campbell^4^, Janice Sinoski^5^, Arlene de la Rocha^6^, Laura Killam^7^

###### ^1^Queen's University (Belfast, Nothern Ireland); ^2^Texas Christian University (Fort Worth, Texas); ^3^University of Ottawa (Ottawa, Ontario, Canada); ^4^University of British Columbia (Vancouver, Canada); ^5^University of Lynchburg (Lynchburg, Virginia); ^6^Ontario Tech University (Oshawa, Canada); ^7^Cambrian College (Sudbury, Canada)

*Advances in Simulation 2025,* **10(1)**:011

**Introduction: context and hypothesis/aims**

As a result of decreasing competency of newly graduated nurses, nursing education leaders are calling for a paradigm shift (1) and competency-based education is gaining momentum to enhance transition of nursing students to practice.(2) Clinical simulation is proposed as a strategy to both build and to assess competence; however, there is limited research supporting how incorporating simulation into nursing programs helps evaluate undergraduate learners’ competency.(3) Furthermore, it is unclear how nursing programs define, measure, and evaluate learners’ competency. This study aims to examine competency definitions, collate current methods for measuring competency, and identify barriers to using simulation to assess learner competency.

**Methods and results: description of the methods used/study design/data collection. Presentation of the results addressing the study hypothesis/aims**

An international team of members of the International Nursing Association for Clinical Simulation and Learning (INACSL) Research Special Interest Group conducted a needs assessment determining current uses and barriers of using simulation to assess competency. Responses from 115 educators from 9 countries spanned pre- and post-licensure academic and hospital training programs. The needs assessment findings guided development of a tool exploring competency evaluation practices for this study. A quantitative cross-sectional descriptive study design was used to survey undergraduate nursing faculty. A multi-method convenience and snowball sampling recruitment strategy was used to obtain an international sample. Survey participants were recruited using QR codes at presentations, discussion board posts, social media, professional organization newsletters, and personal networking. Inclusion criteria required participants to be nurse educators using or planning to use simulation to evaluate undergraduate nursing student competencies.

Eighty-five participants representing 9 countries responded and 70% reported using simulation to evaluate competency. Practices for defining, measuring, leveling, and grading competency varied widely. Participants most often reported measuring clinical judgment (96.6%), communication (93.2%), safety (88.1%), physical assessment (86.4%), and medication administration (86.4%) competencies; 44.8% tracked competency over time. Most commonly reported barriers for measuring competency were workload (28.2%), measuring individuals in groups simulations (23.5%), inadequate staffing (23.5%), tracking performance over time (22.4%), and having insufficient support staff (22.4%). The most common barriers limiting competency evaluations were limited student observation time (54.1%), workload (52.9%), implementing professional development (52.9%), inadequate staffing (51.8%), insufficient space (51.8%), inadequate staff education (51.8%), limited budget (50.6%), multitasking during sim facilitation (50.6%), and believing peer observation changes students’ behavior (50.6%).

**Discussion of the impact/outcome, and novelty of the Research**

Competency definition and assessment criteria variations reflect varied international practice for using simulation to evaluate learners’ competency. Nurse educators need standardized frameworks to ensure learner competency evaluation consistency. Providing better resource allocation, staff training, support, and infrastructure could reduce barriers to assessing learner competency using simulation.

**Keywords**

Nursing education; competency-based education; competency evaluation; clinical simulation

**References/Acknowledgements**

- 1. Kavanagh JM, Sharpnack PA. Crisis in competency: A defining moment in nursing education. Online Journal ofIssues in Nursing. 2021;26(1):2.
- 2. Lewis LS, Rebeschi LM, Hunt E. Nursing education practice update 2022: Competency-based education in nursing. SAGE Open Nursing. 2022;8.
- 3. Cole HS. Competency-based evaluations in undergraduate nursing simulation: A state of the literature. ClinicalSimulation in Nursing. 2023;76:1–16.

## O12. Developing simulation programs in low-resource settings with high medical and humanitarian assistance needs. Médecins Sans Frontières Field Simulation Program in Bobo, Burkina Faso

### Format: Descriptive Work - Oral Presentations, Short Communications and E-posters

#### Topic: Addressing Emerging Healthcare Challenges

##### Marta Iscla, Jeff Mutombo, Laura Castillejos, Carmen Bernal, Alain Kam Sie, Duarte Gomes, Raquel Gaston

###### Médecins Sans Frontières (Geneva, Switzerland)

*Advances in Simulation 2025,* **10(1)**:012

**Introduction: setting, background and identification of needs leading to the initiative**

Médecins Sans Frontières (MSF) provides medical-humanitarian assistance in the Sahel region, where the population is affected by conflicts, epidemics, food insecurity, displacements, and climate change. In this highly unstable and insecure environment, MSF teams face dangerous, stressful conditions with limited capacity for adequate support and training. To address this, MSF is strengthening hospital services in Bobo-Dioulasso, Burkina Faso, to establish a training hub where health professionals from MSF projects in the Sahel can improve their skills in providing medical-humanitarian aid adapted to the needs of the region. Simulation is central to this initiative, providing staff with both: competence in the methodology, and the opportunity to practice and refine their skills in a safe environment. It facilitates the launch of new initiatives, enhances the safety and quality of care, and fosters innovation in these challenging settings.

**Description of initiative and approach/methods used**

MSF initiated the simulation hub in February-2023 to enhance the skills of local and newly recruited staff, and refine hospital's systems, processes, services, and patient care pathways. Experienced simulation staff ensure the program's responsiveness and alignment with ongoing MSF activities. Continuous training of local facilitators in simulation methodology and healthcare topics, such as nursing, pediatrics, neonatology, and sexual and reproductive health, ensures broader coverage and continuous staff training for ongoing quality improvement efforts.

**Discussion of the impact/outcome, and novelty of the initiative**

From February-2023 to August-2024, 11 local simulation facilitators have been trained, 392 simulation sessions have been conducted, reaching 3265 participants in the total number of sessions. Two tabletop and ten on-site simulations, along with regular briefings and debriefings at the workplace, have been completed. The simulation program considered the following factors as contributing to the quality and safety of care and supporting MSF activities: (i) developing staff skills to prevent patients from being the ones testing staff competencies; (ii) testing services to ensure patients are not the ones testing new and existing services, and to identify latent threats; (iii) tabletop simulations to analyze care delays and design effective patient circuits; (iv) clinical debriefings to discuss opportunities for improvement; and (v) briefings and debriefings at shift changes to identify and address challenges collaboratively.

Based on MSF's experience in implementing simulation programs in low-resource settings, it is concluded that to support medical-humanitarian action simulation programs must be implemented in a timely manner, relevant to specific needs, and adaptable to challenging contexts. They should also be readily available with prepared scenarios and facilitators, ensuring accessibility to the simulation for all staff working in these settings.

**Keywords**

Simulation; non-governmental organization; low-resource settings; medical-humanitarian assistance

**References/Acknowledgements**

- Tjoflåt I, Madangi BP, Ralaitafika H, Bø B. Lessons learned through developing and implementing simulation-based education in nursing education programmes in sub-Saharan Africa. International Journal of Africa Nursing Sciences. 2023 Jan 1;19:100592.
- Park-Ross JF, Rowan Duys BE, Gray R, Jansen M. The Simulation Launchpad course: building simulation capacity in Africa. Int. j. healthc. simul. 2024. 10.54531/hfoy7377.
- Van Tetering AA, Ntuyo P, Martens RP, Winter N, Byamugisha J, Oei SG, Fransen AF, van der Hout-van MB. Simulation-based training in emergency obstetric care in sub-Saharan and Central Africa: a scoping review. Annals of Global Health. 2023;89(1).
- Geleto A, Chojenta C, Musa A, Loxton D. Barriers to access and utilization of emergency obstetric care at health facilities in sub-Saharan Africa: a systematic review of literature. Systematic reviews. 2018 Dec;7:1–4.

## O13. Effectiveness of Repeated Standardized Patient Simulations on Self-Efficacy and Attitude Toward End-of-Life Care Among Senior Nursing Students in Türkiye: A Quasi-Experimental Design

### Format: Research Studies - Oral Presentations, Short Communications and E-posters

#### Topic: Culture, Wellbeing, Equity, Diversity, Inclusivity

##### Pinar Dogan, Merve Tarhan, Rabia Eren

###### Istanbul Medipol University (Istanbul, Turkey)

*Advances in Simulation 2025,* **10(1)**:013

**Introduction: context and hypothesis/aims**

Standardized patient simulations have emerged as a valuable method for preparing nursing students to provide end-of-life (EOL) care, enhancing their communication skills and self-efficacy in complex scenarios (1–3). These simulations provide a realistic learning environment for students to engage in challenging patient care conversations and address the emotional dimensions often absent in traditional clinical training (4,5). Previous studies indicate that repeated exposure to EOL simulations improves nursing students'confidence, readiness, and ability to manage sensitive discussions with patients and families (5,6). Furthermore, incorporating such training into nursing curricula can bridge the gap between theoretical knowledge and practical skills, ultimately better-preparing students for real-world care situations (3,5). This study explored the effectiveness of repeated standardized patient simulations on the attitudes and self-efficacy of senior nursing students in Türkiye.

**Methods and results: description of the methods used/study design/data collection. Presentation of the results addressing the study hypothesis/aims**

A quasi-experimental design with a control group was conducted with 77 senior nursing students at a private, accredited nursing program in Istanbul, Türkiye. The intervention group participated in a simulation, and the control group experienced three simulations. Personal information form, EOL and Postmortem Self-Efficacy Scale, and Frommelt Attitudes Toward Care of the Dying Scale were used for data collection. This study was approved by an ethics review committee (Decision Number: 247, Date: 29.02.2024). There was a statistically significant improvement in the intervention and control groups between the pre-test and post-test for self-efficacy (p<0.001; p<0.001) and positive attitudes (p<0.001; p<0.05). The self-efficacy of the intervention group was statistically higher than that of the control group in the post-test (p<0.05). There was no statistical difference in the intervention and control groups for attitudes in the post-test (p>0.05).

**Discussion of the impact/outcome, and novelty of the Research**

The study results demonstrated that repeated standardized patient simulations significantly enhance self-efficacy in EOL care, which aligns with previous studies (1,5). The heightened self-efficacy implies that repetitive exposure aids students in feeling more adept at managing intricate care scenarios, ultimately fostering greater confidence in real-life settings (2,3). Nevertheless, there was no significant difference in attitudes toward EOL care, indicating mixed results observed in prior studies where affective outcomes were more challenging to influence through simulation alone (4,5). This underscores the potential necessity of further integrating complementary educational strategies, such as reflective debriefing or mentoring, to influence attitudes (6). The study's novelty lies in its emphasis on repeated standardized patient interactions. These interactions offer a realistic, dynamic learning experience that equips students for EOL care better than single or less immersive simulations.

**Keywords**

End-of- life care, nursing students, simulation-based learning

**References/Acknowledgements**

1. Steinacker AC, Kreiss V. Can death be simulated? Teaching end-of-life care with simulation in nursing education. JPalliat Med. 2021; 24(9): 1152–1158
2. Lindberg E, Fridh I. Postgraduate nursing students'experiences of simulation training and reflection in end-of-lifecommunication with intensive care patients and their families. Nursing & Health Sciences, 2021; 23(4): 852–861.
3. Escribano S, Cabañero-Martínez MJ, Fernández-Alcántara M, García-Sanjuán S, Montoya-Juárez R, Juliá-Sanchis R. Efficacy of a standardised patient simulation programme for chronicity and end-of-life care training in undergraduate nursing students. Int J Environ Res Public Health, 2021; 18(21): 11,673.
4. Hall K, Bhowmik J, Simonda I, Edward KL. The use of simulated participant and virtual reality simulation to enhancenursing students’ communication skills in “end of life care”—A single-arm repeated measures study. Clin Simul Nurs. 2024; 91: 101,543.
5. Dias R, Robinson K, Poirier P. The effect of simulation on nursing student perceptions of readiness to provideend-of-life care. Journal of Hospice & Palliative Nursing, 2023; 25(6): 116–123.
6. Daly S, Roberts S, Winn S, Greene L. Implementation and evaluation of an end-of-life standardized participantsimulation in an adult/gerontology acute care nurse practitioner program. Nurs Educ Perspect. 2024; 45(3): 172–173

## O14. Enhancing Technical Skills in Nursing Education Through Mental Simulation: A Case Study Using the PETTLEP Model

### Format: Research Studies - Oral Presentations, Short Communications and E-posters

#### Topic: Curriculum Development and Assessment

##### Deborah Hilderson, Geert Van de Weyer

###### KdG University of Applied Sciences and Arts (Antwerp, Belgium)

*Advances in Simulation 2025,* **10(1)**:014

**Introduction: context and hypothesis/aims**

Simulation-based education is a well-established concept in undergraduate nursing education (1). However, simulation can also be applied using a tool everyone possesses: the brain (2). Mental simulation involves consciously visualizing professional scenarios like a ‘mind movie’ (2). This strategy, already used in elite sports and high-risk professions to enhance performance, is also applicable in simulation-based education. Mental simulation is an effective learning strategy that can be applied anywhere. It involves creating purposeful mental representations using all senses and evoking emotions (2). This cognitive practice of motor skills, without physical movement, can improve technical skills and boost self-confidence (2). In nursing education, mental simulation can be used by instructors to enhance the execution of technical skills. Essential to mental simulation is the use of imagery (1), which increases mental resilience and performance under acute pressure and can reduce stress levels (2). It adds value to learning technical procedures, such as those in undergraduate nursing programs.

**Methods and results: description of the methods used/study design/data collection. Presentation of the results addressing the study hypothesis/aims**

A case study and the development of a mental simulation protocol based on the PETTLEP model (3) is presented. The PETTLEP model outlines seven elements essential for effective mental simulation: Physical, Environment, Task, Timing, Learning, Emotion, and Perspective. By repeatedly practicing a technique or procedure mentally, the brain simulates reality, making no distinction between actual performance and visualization. A detailed protocol for mental simulation was developed, including steps such as writing a success story, observing the examination room, performing breathing exercises and creating a safe learning environment. Students were guided by instructors during the mental simulation and received instructions for both preparation and execution of nursing procedures, such as nasogastric tube placement.

**Discussion of the impact/outcome, and novelty of the Research**

Mental simulation seems to be an effective tool for improving technical skills of nursing students. It provides a safe and controlled environment where students can practice complex technical skills and build confidence. While mental simulation is just one of many strategies, it should be integrated with other methods such as hands-on practice and traditional simulation exercises to ensure a comprehensive approach to prepare nursing students into clinical practice.

**Keywords**

Mental simulation - mental rehearsal - case study

**References/Acknowledgements**

1. Roghayeh, Mehdipour-Rabori., Behnaz, Bagherian., Monirsadat, Nematollahi. 6. Simulation-based mastery improvesnursing skills in BSc nursing students: a quasi-experimental study. BMC Nursing, (2021). 10.1186/s12912-020-00532-9.
2. Dimitrios, Stefanidis., Dimitrios, Stefanidis., Nicholas, E., Anton., Graham, McRary., Lisa, D., Howley., Manuel,Pimentel., Cameron, K., Davis., Ashley, M., Yurco., Nick, Sevdalis., Charles, Brown. 7. Implementation results of a novel comprehensive mental skills curriculum during simulator training. American Journal of Surgery, (2017). 10.1016/j.amjsurg.2016.06.027.
3. Holmes, P. S., & Collins, D. J. (2001). The PETTLEP Approach to Motor Imagery: A Functional Equivalence Model for Sport Psychologists. Journal of Applied Sport Psychology, 13(1), 60–83. 10.1080/10413200109339004.

## O15. Exercise Esplanade: Combining tabletop simulation with lab scenarios to train Emergency Department clinicians how to respond to a mass casualty event

### Format: Descriptive Work - Oral Presentations, Short Communications and E-posters

#### Topic: Interprofessional/Team Education and Training

##### Melissa Watts, Jane Vickery, Tracey Bhar, Holly Criddle

###### Fiona Stanley Fremantle Hospitals Group (Murdoch, Western Australia)

*Advances in Simulation 2025,* **10(1)**:015

**Introduction: setting, background and identification of needs leading to the initiative**

It is vital for Emergency Departments (EDs) to practice preparedness for mass casualty events to ensure an effective response (1). Simulation is a powerful tool to assist in staff readiness training (2). However, faculty face challenges when trying to apply mannequin-based simulation activities to disaster response - we do not have enough time, space or equipment (3). Tabletop exercises are commonly used for this purpose, but learners may struggle to immerse themselves in a more hypothetical simulation. When asked in 2023 to help educate our medical and nursing staff in mass casualty response, the ED simulation team at Western Australia's Fiona Stanley Hospital got creative.

**Description of initiative and approach/methods used**

Our aim was to educate staff in the ED Code Brown (external emergency) Sub-plan, specifically decanting the ED, casualty reception and disposition planning. Whilst a tabletop exercise would be sufficient to demonstrate these concepts, we felt that learning in relation to role allocation and prioritisation could be enhanced by immersing participants in a scenario that involved the management of critically unwell patients arriving simultaneously. We decided to combine the two forms of simulation to our learners'benefit.

We used the format of a half-day workshop. As our hospital houses the State Adult Burns Service, we planned an incident involving a hotel fire (Hotel Esplanade, hence the exercise name). Thirty-one ED medical (predominantly registrars) and nursing staff participated. For the tabletop exercise, participants moved between a series of whiteboards containing the ED floor plan to decant patients, then collaborated to allocate staff and resources to casualties. In the simulation lab, participants received two major burns patients simultaneously. Participants had to decide how best to allocate staff and equipment to maximise care. Scenarios were followed by debriefs, in line with Kolb's learning cycle (4).

Collaboration with our Burns Service, anaesthetics department, Disaster Preparedness and Management unit, and Safety and Incident Management Service was crucial to planning. These stakeholders were embedded participants and content experts in the exercises.

**Discussion of the impact/outcome, and novelty of the initiative**

Post-exercise feedback included:

"Very useful in terms of disaster planning and surge planning."

"Amazing learning opportunity."

"excellent to get first hand experience...really enjoyed having a dual sim at same time to split resources!"

We were also able to identify issues with the Sub-Plan. This led to several improvements, including clarification on key roles for after-hours use and alterations to action role cards.

This exercise serves to highlight that combining different styles of simulation (ie. a more hypothetical activity with mannequin-based scenarios) can help to enhance the learning experience for mass casualty incidents. There is scope to apply this model to other clinical scenarios such as pandemic preparedness.

The exercise is run annually.

**Keywords**

Table top, emergency, emergency medicine, mass casualty, disaster

**References/Acknowledgements**

**References**

1. Australasian College for Emergency Medicine. Emergency department disaster preparedness and response Policyv02 [Internet]. Melbourne (VIC): ACEM; 2020 [cited 2024 Oct 20]. 11p. Policy No.: P33. Available from: https://acem.org.au/getmedia/f955b382-891c-46d1-aaf6-11f9a695ee35/Policy_on_ED_Disaster_Preparedness_and_Response.
2. Kerner RL, Gallo K, Cassara M, D'Angelo J, Egan A, Simmons JG. Simulation for Operational Readiness in a NewFreestanding Emergency Department: Strategy and Tactics. Simul Healthc. 2016 Oct;11(5):345-56.
3. Moss R, Gaarder C. Exercising for mass casualty preparedness. Br J Anaesth. 2022;128(2):e67-e70.
4. Fanning RM, Gaba DM. The Role of Debriefing in Simulation-Based Learning. Simul Healthc. 2007;2(2):115-25.

**Acknowledgements**

Dr Luke Pritchard, FSH Emergency Department

Sophie Barker, FSH Emergency Department

Mike Hayward, Area Manager, Disaster Preparedness and Management, South Metropolitan Health Service

Helen Barrett, Business Continuity Lead, Disaster Preparedness & Management, Fiona Stanley Fremantle Hospitals Group

Piyush Piyush, Emergency Management Advisor, Safety and Incident Management Service, Fiona Stanley Fremantle Hospitals Group

Major Rachel Howes, Burns Fellow, Fiona Stanley Fremantle Hospitals Group

Dr Suzanne Rea, Consultant Burns Surgeon, Fiona Stanley Fremantle Hospitals Group Dr Helen Douglas, Consultant Burns Surgeon, Fiona Stanley Fremantle Hospitals Group 

## O16. Exploring the Landscape of Simulation-based Education in Emergency Medical Services (ELSE—EMS) – Development of a global survey by the SESAM Pre-Hospital Community of Practice

### Format: Descriptive Work - Oral Presentations, Short Communications and E-posters

#### Topic: Quality assurance, Faculty development and Program evaluation

##### Vitor Almeida^1^, Kenneth Boe Krarup^2^, Guillaume Alinier^1^, Thomas Marian^1^, G. Ulufer Sivrikaya^1^

###### ^1^Society in Europe for Simulation Applied to Medicine (SESAM) Pre-Hospital Community of Practice (PH CoP); ^2^Odense Teaching Hospital, Region of southern Denmark

*Advances in Simulation 2025,* **10(1)**:016

**Introduction: setting, background and identification of needs leading to the initiative**

Emergency Medical Services (EMS) are globally characterised by a variety of legal and organizational aspects. Education and continuous training in an interprofessional approach are key aspects in EMS performance. It is well known that substantial heterogeneity exist for requirements, training and continuous professional development of the personnel (1–3). This variability is generally aligned with the varied scope of practice among EMS professionals within and across nations, depending on their professional title and is affected by the system in which these professions operate within a legal framework. This poses significant challenges that may have impact in patient safety, the efficiency and effectiveness of EMS systems, and requires to be investigated. The aim is to promote the optimal adoption of simulation-based education (SBE) and elaborate SESAM PH-COP recommendations. Despite its recognized advantages and widespread use, there is a lack of comprehensive knowledge regarding the level of adoption of SBE for EMS professionals, the types of simulation activities and modalities they get exposed to, requirements for trainers and educational facilities, as well as legal mandatory frameworks.

**Description of initiative and approach/methods used**

The Pre-Hospital Community of Practice of the Society for Simulation in Europe (SESAM PH CoP) decided to develop a survey using a Delphi process to elucidate this question in greater detail. The survey was developed through online meetings over a 18 months period and involved a multi-professional team of pre-hospital care educators. The survey will be hosted on Microsoft Forms and shared via socialmedia platforms and through the networks of the SESAM PH CoP and other ScientificSocieties. The target sample is anyone involved in the education of EMS students or professionals and the survey will remain open for completion for 6 months.

The objectives of the survey are to determine: (1) if there are associations between SBE methodologies, tools, and practices used and the professions of EMS personnel being trained, and their type of organisation; (2) if the educators and organisations have any form of recognised SBE certification or accreditation; (3) if the respondents’ perceive SBE to be an effective training approach; (4) the perceived level of buy-in of the participants’ organisation in incorporating SBE in their training programmes.

**Discussion of the impact/outcome, and novelty of the initiative**

Rather than always remaining in their comfort zone, it is important for educators to reflect on their educational practices and consider engaging with new approaches, tools, and standards which are proven to be more effective in helping learners to acquire and maintain new skills and knowledge. Even if it is solely based on a convenience sample of participants which may introduce some level of selection bias, the outcome of the survey will provide benchmarking information for Global EMS training organisations that may contribute to the standardization and increase of the quality of SBE programmes, and therefore contribute to a improved harmonization of EMS Systems.

**Keywords**

Prehospital Care, Emergency Medical Systems, Ambulance Service

**References/Acknowledgements**

1. Bentley MA, Shoben A, Levine R. The demographics and education of emergency medicalservices (EMS) professionals: a national longitudinal investigation. Prehospital and disaster medicine. 2016 Dec;31(S1):S18-29.
2. Demir S, Tunçbilek Z, Naidoo V, Morris T, Alinier G. Paramedic education in Qatar as seen byacademics from Turkey. International Paramedic Practice. 2023 Feb 2;13(1):2–8.
3. Eisner ZJ, Diango K, Sun JH. Education and training of prehospital first responders in low-andmiddle-income countries. Surgery. 2024 Jul 1;176(1):226–9.

## O17. Impact of Simulation Training on Communication in the Surgical Handover

### Format: Research Studies - Oral Presentations, Short Communications and E-posters

#### Topic: Curriculum Development and Assessment

##### Cathleen A. McCarrick, Philip D. McEntee, Patrick A. Boland, Suzanne Donnelly, Helen Heneghan, Ronan A. Cahill

###### University College Dublin (Dublin, Ireland)

*Advances in Simulation 2025,* **10(1)**:017

**Introduction: context and hypothesis/aims**

Effective communication skills are integral to a physician's practice, particularly in high-stakes environments like surgical handover. This study explores the innovative application of Communication Simulation Training (CST) using the ISBAR tool to enhance these critical skills among final-year medical students. While ISBAR is widely recognized for its role in ensuring clear communication and patient safety, our approach uniquely integrates simulation-based learning with peer reenactment and discussion, which has not been extensively studied in this context. By assessing the impact of CST on students'communication abilities, we aim to provide new insights into how structured training can improve surgical handover practices and contribute to the development of competent future healthcare professionals. In this study, we designed, implemented, and evaluated a Communication Simulation Training (CST) program aimed at enhancing surgical handover skills among final-year medical students.

**Methods and results: description of the methods used/study design/data collection. Presentation of the results addressing the study hypothesis/aims**

With institutional ethics approval, CST was integrated into our undergraduate clinical surgery module. Students were randomly divided into two groups, with all participants already familiar with the ISBAR framework. Before the intervention, each student was assessed on their communication skills using the externally validated Global Communication Rating Scale (GCRS) and adherence to ISBAR during a mock surgical handover, evaluated by an independent blinded senior clinician. Control students continued with standard clinical experiential learning, while the intervention group participated in CST, which included observing effective and ineffective surgical handover roleplays using ISBAR, followed by peer reenactment and discussion. Both groups underwent repeat assessments in the same week. Additionally, intervention group students completed surveys to anonymously report their confidence levels in communication before and after CST.

A total of 132 students participated, with 68 undergoing CST. Mean GCRS scores were similar (p > 0.05) at baseline but significantly higher for CST-trained students (p < 0.01). Improvements were particularly notable in the GCRS domains of initiation, verbal communication, session structuring, and information relay. Self-reported confidence levels also increased significantly (2.93 vs. 3.96; p < 0.05, >80% response rate).

**Discussion of the impact/outcome, and novelty of the Research**

Communication Simulation Training significantly enhances students'communication abilities, particularly in surgical handover. This study is unique in its approach, combining CST with peer reenactment, which effectively reinforces learning and skill retention. The skills developed are transferable to other communication contexts, ensuring that soon-to-be-qualified doctors are proficient in articulating surgical patient situations effectively, ultimately leading to improved patient care and safety.

**Keywords**

Surgery, Simulation, Handover, Communication

**References/Acknowledgements**

- Burgess A, van Diggele C, Roberts C, Mellis C. Teaching clinical handover with ISBAR. BMC Med Educ. 2020 Dec 3;20(Suppl 2):459.

## O18. Impact of a Simulation-Based Learning Program on Clinical Skills and Patient Outcomes in Transthoracic Echocardiography

### Format: Research Studies - Oral Presentations, Short Communications and E-posters

#### Topic: Quality assurance, Faculty development and Program evaluation

##### Jordi Baneras^1^, Michelle Laurens Acevedo^1^, Sofía Contreras^1^, Ignasi Maspons^1^, Monica Rodriguez-Carballeira^1^, Laura Galian^2^, Gerard Camp^2^, Angel Vazquez^2^, Filipa Rosa Xavier de Carvalho Negrão Valente^2^, Jose Rodriguez-Palomares^2^, Ignacio Ferreira^3^

###### ^1^Vall d´ Hebron Advanced Clinical Simulation Center (Barcelona, Spain); ^2^Cardiovascular Image-Hospital Vall Hebron (Barcelona, Spain); ^3^Hospital Vall Hebron (Barcelona, Spain

*Advances in Simulation 2025,* **10(1)**:018

**Introduction: context and hypothesis/aims**

Mastery of transthoracic echocardiography (TTE) is essential for physicians across several specialties, yet it is often hindered by limited clinical exposure and concerns about patient comfort. Simulation-based education provides an effective alternative by offering a controlled, risk-free learning environment. The [ECOCARSIM] program was designed to integrate simulation into TTE training for residents, aiming to improve technical skills and confidence. This study hypothesizes that simulation-based training will enhance both resident proficiency in TTE and patient experience compared to traditional methods.

**Methods and results: description of the methods used/study design/data collection. Presentation of the results addressing the study hypothesis/aims**

In this observational study, 60 residents from cardiology, anesthesiology, intensive care, internal medicine, and neurology at Hospital Universitari Vall d’Hebron, Barcelona, were divided into two groups: Simulation-Trained (n=30, ECOCARSIM) and Traditionally Trained (n=30). The ECOCARSIM program combined theoretical online modules and practical sessions using high-fidelity simulators. The primary outcomes were image quality scores (0–10 points per view) and examination time. Secondary outcomes included patient experience (assessed via a visual analog scale) and resident satisfaction and confidence (measured using the Student Satisfaction and Self-Confidence in Learning Scale).

The Simulation-Trained group achieved significantly higher image quality scores across all echocardiographic views (3.28±2.98 to 6.79±3.35, p<0.001). No significant difference in examination time was observed between the groups (10.53±3.5 vs. 11±4.1 minutes, p=0.36). Patients examined by Simulation-Trained residents reported less discomfort (VAS: 0.89±1.84 vs. 1.36±1.51, p=0.043). Resident satisfaction scores were notably higher in the Simulation-Trained group (4.37 vs. 3.32, p=0.007), as were confidence levels (4.46 vs. 3.83, p=0.013).

**Discussion of the impact/outcome, and novelty of the Research**

The findings demonstrate that the ECOCARSIM simulation-based training program significantly enhances residents’ technical proficiency and confidence in performing TTE. Furthermore, the program improves patient comfort without increasing the time required to complete the procedure. This research contributes novel evidence supporting the integration of simulation-based learning into echocardiography training programs, showing clear benefits for both clinical skills development and patient care. The use of simulation to achieve high-quality image acquisition and improve resident confidence represents an innovative step in modernizing medical education for the specialties requiring TTE skills.

**Keywords**

Transthoracic Echocardiography, Simulation-Based Learning, Clinical Skills Training, Patient Outcomes

**References/Acknowledgements**

- Frederiksen CA, Juhl-Olsen P, Nielsen DG, Sloth E. Limited intervention improves technical skill in focused cardiac ultrasonography for non-cardiologists. Eur J Anaesthesiol. 2012;29(4):157–161.
- Mankad S, Bonnichsen CR, Pellikka PA. The role of simulation in echocardiography training: A narrative review. J Ultrasound Med. 2016;35(11):2305-2311.
- Biswas M, Patel R, German C, et al. Simulation-based training in echocardiography. Echocardiography. 2016;33(10):1581-1588.

**Fig. 1 (O18):**
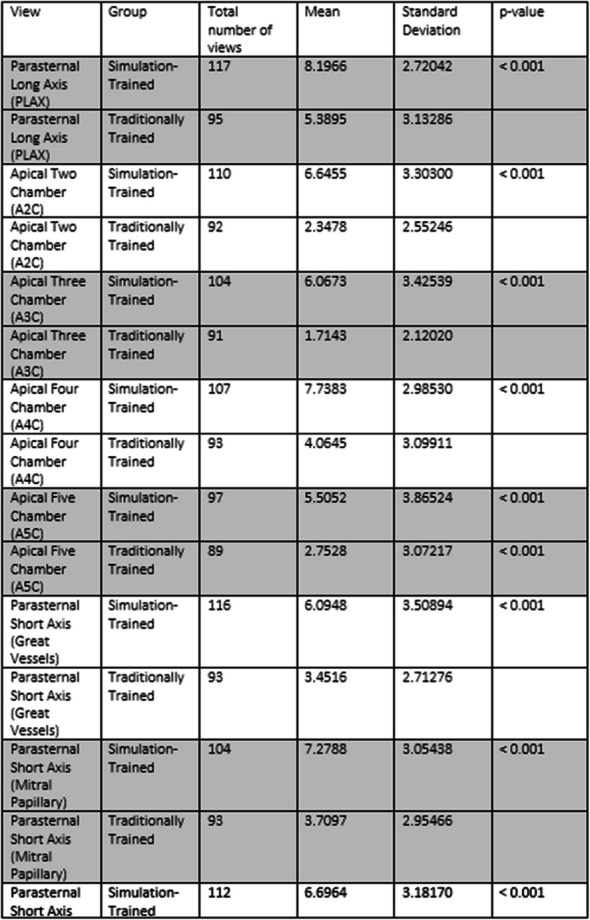
see text for description

**Fig. 2 (O18):**
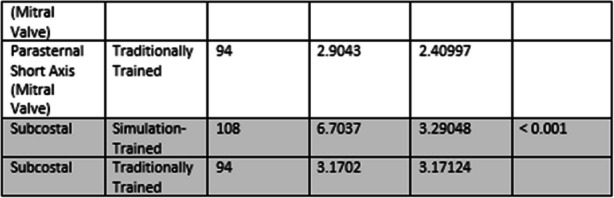
see text for description

## O19. Improving access to practical training using low-cost models: insights from Médecins sans Frontières

### Format: Descriptive Work - Oral Presentations, Short Communications and E-posters

#### Topic: Addressing Emerging Healthcare Challenges

##### Irene Perez-Garcia^1^, Marta Iscla-Aragones^2^, Griselda Gonzalez-Caminal^3^

###### ^1^Simulation Mobile Implementation Officer (Barcelona Spain); ^2^Field Simulation Program Manager (Barcelona Spain; ^3^Simulation Capacity Building Technician (Barcelona Spain)

*Advances in Simulation 2025,* **10(1)**:019

**Introduction: context and hypothesis/aims**

The humanitarian contexts in which Médecins Sans Frontières (MSF) works are fragile, challenging, and complex, requiring great flexibility and adaptability of action. MSF works to provide quality, safe, and people-centered healthcare. In addition to system and process improvement, MSF uses simulation to train competencies. The immediate availability of low-cost equipment to practice specific techniques must be ensured, although it might be challenging due to the abovementioned barriers. Therefore, different departments in MSF are investing in creating low-cost training models assembled from materials present in the projects as an operational and sustainable solution. This work presents and validates a prototype for a neonatal lumbar puncture model (NLP), analyzed in terms of fidelity and usefulness used to train staff in a neonatology department in Afghanistan.

**Methods and results: description of the methods used/study design/data collection. Presentation of the results addressing the study hypothesis/aims**

A prototype1 for NLP has been developed using an adapted rapid prototyping model to test and evaluate by means of an ad hoc questionnaire (2) consisting of three dimensions: (i) fidelity (visual and tactile appearance) (ii) usability and (iii) general assessment of the model; using a Likert-type scale from 1 (worst) to 10 (best). The prototype1 is assembled with seven out of nine elements present in the project and has been tested by 26 people (23 participants and three experts). Fidelity and usability outcomes were highly rated from both, participants and experts obtaining scores >8 over 10. The global assessment of the model was equally highly rated (see Figure 1).

**Discussion of the impact/outcome, and novelty of the Research**

Preliminary data show significant acceptance for NLP prototype1. Nevertheless, more practice is needed to improve and refine the prototype. This lumbar puncture model exemplifies one of the many low-cost models assembled to improve the skills of MSF field staff where people with limited simulation resources. Most importantly, MSF has and continues to develop low-cost models in different areas: sexual and reproductive care, basic technical skills such as intraosseous and suturing, among others. Those models are aligned with sustainability principles using material present in each project and ensuring immediate accessibility to specific and necessary staff training. Considering MSF context, the significance of comprehensive training cannot be overstated, especially for local staff who have been disadvantaged lack of opportunities to safely practice, using simulators, invasive techniques and procedures.

**Keywords**

Simulation; NGO; low-cost trainers; rapid prototyping; humanitarian settings.

**References/Acknowledgements**

- Costa R, Araújo M, de Medeiros S, Mazzo A. Development and Content and Face Validation of Low-Cost Simulators Evaluation Instrument. Clinic Sim Nurs. 2024, 91(2):101539
- López-Baamonde M, Perdomo J.M, Ibáñez C, Angelès-Fité G, Magaldi M, Panzeri M.F, Bergé R, Gómez-López L, Guirao Montes Á, Gomar-Sancho C, & SIMCLÍNIC-ANESTHESIOLOGY. Construction and Evaluation of a Realistic Low-Cost Model for Training in Chest-Tube Insertion. Sim healthc. 2024, 19(3),188–195.
- Martin J. Rapid application development. Indianapolis: Macmillan Publishing Co., Inc; 1991.

**Fig. 1 (O19):**
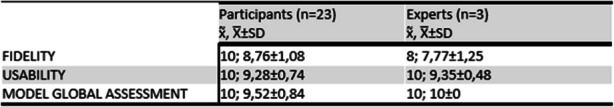
Results of prototype 1 by means of fidelity and usability of a NLP model

## O20. Influence of arm angle on chest compression depth and muscle fatigue: Insights from an international, multicentric, randomised simulation study

### Format: Research Studies - Oral Presentations, Short Communications and E-posters

#### Topic: Patient Safety and Quality Improvement

##### Carla Sá-Couto^1^, Ingrid Bispo^1^, Abel Nicolau^1^, Pedro Vieira-Marques^1^, Marc Lazarovici^2^, Christoffer Ericsson^3^

###### ^1^Faculty of Medicine, University of Porto (FMUP) (Porto, Portugal); ^2^LMU University Hospital (München, Germany); ^3^University of Helsinki, Faculty of Medicine (Helsinki, Finland)

*Advances in Simulation 2025,* **10(1)**:020

**Introduction: context and hypothesis/aims**

During chest compressions (CC), rescuers may adjust their posture as fatigue develops, such as leaning backwards and altering the arm angle relative to the victim's chest [1, 2]. Such adjustments may decrease CC quality and increase rescuer fatigue, indicated by changes in muscle contraction frequency [3]. This study aims to assess the influence of arm angle on CC quality and muscle fatigue.

**Methods and results: description of the methods used/study design/data collection. Presentation of the results addressing the study hypothesis/aims**

**Methods**

An international, multicentre, randomised simulation study was conducted in Portugal, Finland, and Germany, with data collected between May and October 2023. Healthcare professionals experienced in CPR, aged 18–65, were recruited through convenience sampling. Participants were randomised into two groups: (1) kneeling with the manikin on the ground, and (2) standing with the manikin on a bed adjusted to knee height (no mattress used to prevent CC damping). Each participant performed two 3-minute CC sessions at arm angles of 90° and 105°, with a 10-minute rest between trials. CC depth assessed CC quality, and perceived exertion was evaluated using the Borg Scale after each trial. CC waveform data were captured by the simulator (Laerdal SimPAD) and processed using MATLAB to extract CC depth. Electromyogram (EMG) electrodes were placed on both triceps brachii to assess muscle contraction during CC. EMG data were processed using MATLAB, including filtering, activation detection, and computation of instantaneous median frequency (iMDF). The area under the iMDF curve (AUC-IMDF) was calculated using numerical integration.

Sociodemographic data were collected via questionnaire. Ethical approval was obtained prior to the study.

**Results**

Group 1 (kneeling) included 21 individuals (mean age 37 ± 10.7, 48% female), and Group 2 (standing) had 25 participants (mean age 39 ± 11.4, 60% female). After performing CC with a 105° arm angle, both groups reported higher exhaustion (Borg Scale median 15 vs. 13, p < 0,001) and were unable to maintain adequate CC depth (p < 0,001). Greater muscle fatigue was also observed, indicated by lower muscle contraction frequency compared to the 90° angle (p < 0,001). Figure 1 presents the CC depth and iMDF data sampled every 15 seconds, along with boxplots of the AUC-iMDF, providing an integrated view of the results.

**Discussion of the impact/outcome, and novelty of the Research**

Performing CC with a 105° arm angle led to increased muscle fatigue, higher reported exhaustion, and decreased CC depth compared to the standard 90° arm angle. These results suggest that posture significantly influences muscle fatigue and CC quality.

**Keywords**

Cardiopulmonary resuscitation (CPR); chest compressions quality; muscular fatigue; electromyography (EMG)

**References/Acknowledgements**

1. Foo NP, Chang JH, Lin HJ, Guo HR. Rescuer fatigue and cardiopulmonary resuscitation positions: A randomizedcontrolled crossover trial. Resuscitation. 2010;81(5):579–84.
2. Sato N, Karino K, Hirose M, Okamoto S, Osaka T, Matsumura H, et al. Chest compressions become deeper whenpushing with forward lean: A simulation study. Resusc Plus. 2021 Oct 13;8:100,169. https://doi.org/10.1016/j.resplu.2021.100169.
3. Allison GT, Fujiwara T. The relationship between EMG median frequency and low frequency band amplitude changesat different levels of muscle capacity. Clin Biomech (Bristol, Avon). 2002;17(6):464–9.

**Fig. 1 (O20):**
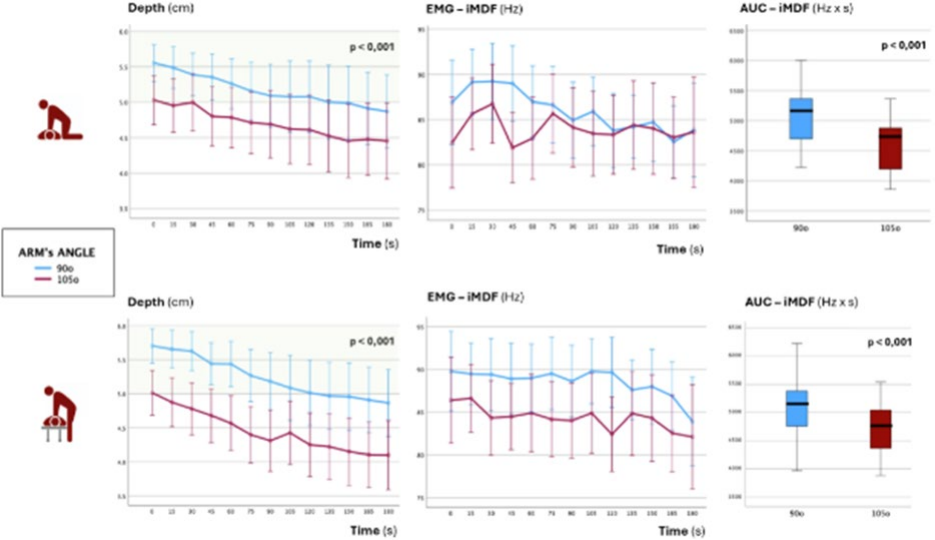
Changes in quality and muscle contraction over 3 min of chest compression between 2 arm angles in 2 different rescuer’s positions. cm: centimeters │ s: seconds │ EMG: electromyography │ IMDF: Instantaneous median frequency │ Hz: hertz │ AUC: area under curve

## O21. Investigating face emotional reactions during high fidelity simulated difficult intubation learning through deep learning technology

### Format: Research Studies - Oral Presentations, Short Communications and E-posters

#### Topic: Curriculum Development and Assessment

##### Médéric Descoins, Laurent Bocquillon Liger-Belair, Fabien Bruneteau, Clotilde Chevalier, Perrine Levy Valensi

###### CHU de La Réunion (La Réunion - France)

*Advances in Simulation 2025,* **10(1)**:021

**Introduction: context and hypothesis/aims**

In anesthesia training, mastering difficult intubation is essential but challenging, as real-life exposure is limited and stressful. High-fidelity simulation (HF) provides a safe environment for developing these skills. Emotional responses, crucial for learning and memory retention, impact performance in simulated scenarios. By applying a deep learning software : FaceReader® for facial expression analysis, we aim to identify objectively specific emotions with procedural success or failure and examine their potential impact on learning outcomes.

**Methods and results: description of the methods used/study design/data collection. Presentation of the results addressing the study hypothesis/aims**

Conducted at the Indian Ocean Healthcare Simulation Center in January 2023, this prospective, randomized study involved 12 anesthesia trainees (nurses and residents) with limited experience in video laryngoscopy. Each participant performed six HF scenarios with a SimMan® mannequin, alternating between two video laryngoscopes (AirTraq™ and McGrath™). Scenarios included predefined obstacles like trismus and limited neck mobility to simulate difficult airway conditions.

Throughout the simulations, two cameras captured the participants’ faces. Videos were analyzed by FaceReader® software. This tool based on deep learning analysis tracked seven primary emotions (neutral, joy, sadness, disgust, anger, fear, and surprise), tracking changes during critical intubation stages (preoxygenation, induction, trismus, intubation attempt, and post-intubation). Statistical comparisons were performed to assess emotion frequency and intensity at each stage and between the two devices.

Results showed that the"neutral"expression was the most prevalent across all stages (57.31%), followed by sadness and disgust, while joy, surprise, and anger were less frequently observed. The emotion"neutral"was particularly dominant during preoxygenation and post-intubation stages. During failed intubation attempts (n=2; 2.7% of the total realized intubation), participants exhibited significant increases in surprise and sadness, while successful attempts maintained"neutral"expressions. No notable emotional differences were detected between the two laryngoscope types.

**Discussion of the impact/outcome, and novelty of the Research**

This study is the first to use deep learning technology of face video captured in HF simulations to analyze the continuous emotional state of the students during the learning process. The high prevalence of"neutral"expressions may reflect a tendency for emotional suppression or represent a baseline concentration level. The FaceReader® software provides an innovative approach to objective emotional assessment potentially enhancing debriefing processes by allowing instructors to adapt feedback based on real-time emotional data. This research supports the integration of emotional analysis into simulation training, emphasizing its role in both technical and non-technical skill acquisition. However since the European AI Act (into effect on August 1, 2024), this great potential approach is subject to specific regulations.

**Keywords**

Automatic emotion analysis, difficult intubation, Hight-fidelity simulation

**References/Acknowledgements**

- The authors thank all the volunteers, as well as A. Desvergez for assistance in developing the scenario, and HGmedicale Reunion for providing equipment
- LeBlanc VR, Regehr C, Tavares W, Scott AK, MacDonald R, King K. The Impact of Stress on Paramedic Performance During Simulated Critical Events. Prehospital Disaster Med. 2012;27(4):369–374. https://doi.org/10.1017/S1049023X12001021
- Harvey A, Bandiera G, Nathens AB, LeBlanc VR. Impact of stress on resident performance in simulated trauma scenarios. J Trauma Acute Care Surg. 2012;72(2):497–503. https://doi.org/10.1097/TA.0b013e31821f84be
- Madsgaard A, Smith-Strøm H, Hunskår I, Røykenes K. A rollercoaster of emotions: An integrative review of emotions and its impact on health professional students’ learning in simulation-based education. Nurs Open. 2022;9(1):108–121. https://doi.org/10.1002/nop2.1100

## O22. Mental Health Simulation for Specilaised Mental Health Paramedics

### Format: Descriptive Work - Oral Presentations, Short Communications and E-posters

#### Topic: Addressing Emerging Healthcare Challenges

##### Naomi Tomlinson, Elaine Thomas, Emma Baxey, Damir Rafi, Anita Bignell, Antonia Winney, Ermias Alemu, Megan Fisher, Sam Ter Horst, Selena Galloway, Hannah Iannelli, Lloyd Campbell

###### Maudsley Learning (London, United Kingdom)

*Advances in Simulation 2025,* **10(1)**:022

**Introduction: setting, background and identification of needs leading to the initiative**

Ambulance services in the UK are encountering mental health presentations of increasing frequency and complexity 1. Reasons for this include greater demand on psychiatric inpatient beds, longer waiting lists for community treatment and the recent nationwide adoption of the Right Care, Right Person operational model 2. As set out in the NHS Long Term Plan, many ambulance services have created new roles for Specialist Mental Health Paramedics (SMHP’s) 3. However, there is no standardised curriculum for how to train and support these professionals.

**Description of initiative and approach/methods used**

We were initially commissioned by a regional ambulance service to design and provide a bespoke five-day training package for 24 new SMHP’s. This course combined four days of online learning (covering subjects including reducing restrictive practice, mental health legislation and risk-assessment) and one day of live-delivery simulation. We delivered the simulation on two consecutive days in November 2023, for two groups of 12 new SMHP’s. Six scenarios, played by two professional actors, were debriefed by two faculty members and a subject matter expert (a senior paramedic). The simulation day was unanimously rated as excellent, and widely cited as the most helpful aspect of the course, as an “immersive [chance to] consolidate knowledge.” Pre and post course questionnaires showed increased confidence levels across all the learning objectives (see Figure 1).

Following this success, we were re-commissioned to provide a one-year refresher simulation day, for the same group who were now one year into their new roles. The course followed the same format, with the same learning objectives carried through from the original days. Feedback was again positive, with increased confidence levels (see Figure 1) and participants commenting on benefitting from space for “personal reflection” and “exploration and challenge of ideas by facilitators.”

**Discussion of the impact/outcome, and novelty of the initiative**

We will present the feedback data from both days, and discuss how we developed the complexity of the scenarios from one year to the next; for example, moving from an adult with psychosis to an adolescent, and moving from a new presentation of self-harm to a frequent attender.

We will explore how free text comments from the first course indicate the benefits of gaining theoretical and practical knowledge and opportunities to practice, while feedback from the second course valued opportunities to reflect on nuances of the scenarios and learn from colleagues. One contributor may be that we moved from a Pendleton’s feedback model to a Maudsley Debrief Model (a modified diamond debrief model).

**Keywords**

Mental Health Simulation, Ambulance Services, Specialised Mental Health Paramedics

**References/Acknowledgements**

- 1. House of Lords - Emergency healthcare: a national emergency - Public Services Committee. Parliament.uk. 2018. Available from: https://publications.parliament.uk/pa/ld5803/ldselect/pubserv/130/13006.htm
- 2. National Partnership Agreement: Right Care, Right Person. GOV.UK. Available from: https://www.gov.uk/government/publications/national-partnership-agreement-right-care-right-person
- 3. NHS Mental Health Implementation Plan 2019/20–2023/24. https://www.longtermplan.nhs.uk/wp-content/uploads/2019/07/nhs-mental-health-implementation-plan-2019-20-2023-24.pdf. NHS England; 2019 [cited 2024 Oct 20]. Available from: https://www.longtermplan.nhs.uk/wp-content/uploads/2019/07/nhs-mental-health-implementation-plan-2019-20-2023-24.pdf

**Fig. 1 (O22):**
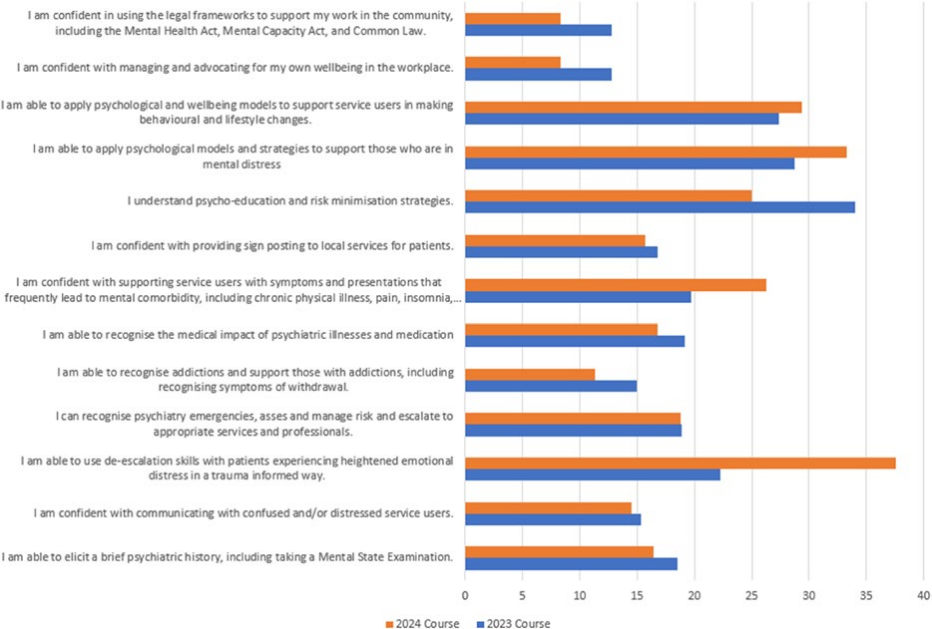
Percentage Increase in Confidence from Pre-course to Post-course Questionnaires

## O23. Pulling back the curtain: exploring the inner dynamics of impostor phenomenon in simulation educators

### Format: Research Studies - Oral Presentations, Short Communications and E-posters

#### Topic: Quality assurance, Faculty development and Program evaluation

##### Kirsty J Freeman^1,2^, Sandra E Carr^2^, Stephen Houghton^2^, Debra Nestel^3^

###### ^1^The Rural Clinical School of Western Australia (Perth, Australia); ^2^The University of Western Australia (Perth, Australia); ^3^Monash University (Melbourne, Australia)

*Advances in Simulation 2025,* **10(1)**:023

**Introduction: context and hypothesis/aims**

Impostor phenomenon (IP) is a persistent experience characterised by self-doubt, anxiety, and the fear of being exposed as a fraud, despite external validation of competence(1, 2). This qualitative study explores how healthcare simulation educators experience and navigate IP throughout their careers, providing insight into how it influences their professional identity and career trajectories. By focusing on the complex, cyclical nature of IP, this research addresses the broader implications of impostorism within professional environments and offers valuable guidance for the healthcare simulation community.

**Methods and results: description of the methods used/study design/data collection. Presentation of the results addressing the study hypothesis/aims**

A hermeneutic phenomenological approach (3) was employed to explore the lived experiences of 20 healthcare simulation educators who self-reported significant impostor feelings via the Clance Impostor Phenomenon Scale (CIPS)(4). Semi-structured interviews were conducted, transcribed, and analysed iteratively, following the principles of hermeneutic inquiry. Through reflexive engagement and collaborative team discussions, four key themes were identified:

- I Don’t Have the Right Badges: Participants expressed a need for external validation through qualifications andprofessional recognition.
- Now You See Me, Now You Don’t: Educators described minimising their professional presence, fearing exposure orfailure.
- Friend or Foe: While IP was often a burden, it also served as a motivator for growth.
- Hello, My Old Friend: Participants described the cyclical nature of IP, with feelings of self-doubt resurfacing throughouttheir careers.

**Discussion of the impact/outcome, and novelty of the Research**

This study contributes novel insights into how IP shapes the professional experiences of healthcare simulation educators. It challenges the traditional view of IP as solely detrimental, highlighting its dual role as both an obstacle and a driver for professional development. By pulling back the curtain on the hidden challenges of impostor phenomenon, the findings suggest that tailored interventions, such as mentorship programs and communities of practice, could mitigate the negative impacts of IP, fostering psychological safety and professional resilience.

**Keywords**

Impostor Phenomenon, Professional Identity, Mentorship, Psychological Safety

**References/Acknowledgements**

1. Cokley K, Harris K, Hall S, Singletary M, Cokley K. An overview of the impostor phenomenon: Definitional andTheoretical Considerations. The Impostor Phenomenon. Psychological Research, Theory, and Interventions: American Psychological Association; 2024. p. 45–60.
2. Freeman KJ, Houghton S, Carr SE, Nestel D. Impostor phenomenon in healthcare simulation educators. InternationalJournal of Healthcare Simulation. 2022.
3. Van Manen M. Researching Lived Experience: Human Science for an Action Sensitive Pedagogy. Milton, unitedkingdom: Taylor & Francis Group; 1997.
4. Clance PR, Imes SA. The imposter phenomenon in high achieving women: Dynamics and therapeutic intervention. Psychotherapy (Chicago, Ill). 1978;15(3):241–7.

## O24. Robots to the Rescue: Capacity-busting AI-powered communication simulation to enhance preparedness of medical undergraduates on clinical placements

### Format: Descriptive Work - Oral Presentations, Short Communications and E-posters

#### Topic: Extended Reality, AI and Virtual Simulation Modalities

##### Thom O'Neill, Sarah Galbraith, Sara Robinson

###### NHS Lothian (Edinburgh, Scotland)

*Advances in Simulation 2025,* **10(1)**:024

**Introduction: setting, background and identification of needs leading to the initiative**

Medical student numbers are set to rise dramatically across Europe, including in the UK(1), posing significant capacity challenges to health systems wanting to maintain high-quality education for undergraduates on clinical placements. Key factors affecting student experiences include ‘preparation and preparedness’, ‘confidence and competence’, and ‘supervision and support’ (2).

To enhance preparedness, and improve confidence and competence, NHS Lothian (Edinburgh, Scotland) are trialing an innovative AI-powered communication simulation platform (SimConverse) to deliver custom-built, specialty-specific clinical and communication skills scenarios for medical students entering clinical placements.

**Description of initiative and approach/methods used**

Traditional simulation-based medical education (SBME) supports communication skills training through role-play with standardized patient actors, which is time- and resource-intensive with low repeatability. Whereas the on-demand AI-powered platform is scalable, pairing AI-powered patient characters with detailed, comprehensive AI-powered feedback.

Characters and feedback rubrics are developed by our clinical simulation faculty, with the custom-built generative AI working only within our parameters to facilitate on-demand availability and scalability. Scenarios are built into assignments rooted in experiential learning theory, and supported by pre-briefs for psychological safety. Participants can then repeat scenarios, gaining iterative feedback after each attempt, augmenting the deliberate practice framework(3).

**Discussion of the impact/outcome, and novelty of the initiative**

Full conversation transcripts and comprehensive feedback rubrics can be downloaded for further development through self-reflection or synthesis with in-person tutor discussions and or facilitated debriefs to support learning.

The platform has been shown to increase capacity and decrease educator burden, whilst maintaining the same performance and satisfaction endpoints as traditional SBME(4). Our scenario designs range from clinical history-taking through to difficult conversations and breaking bad news. Virtual platforms can also promote interprofessional communication(5), and we have integrated scenarios involving healthcare professional characters (such as referrals, handovers, and escalations of care) in a way which may also progress systems familiarity as a hidden curriculum.

Our approach aims both to enhance preparedness before placements and augment experiences and skill acquisition during placements for medical undergraduates. By providing additional opportunities for iterative rehearsal of clinical histories and communication skills with immediate meaningful feedback, we aim to use this platform as a tool to help address capacity challenges in a scalable and engaging manner. Initial feedback from students has been excellent, and comprehensive research on both student and supervisor experiences using this platform is underway.

**Keywords**

Artificial intelligence, medical education, communication, simulation, deliberate practice, experiential learning, undergraduate capacity

**References/Acknowledgements**

1. The expansion of medical student numbers in the United Kingdom Medical Schools Council Position Paper [Online]. 2021. Available from: https://www.medschools.ac.uk/media/2899/the-expansion-of-medical-student-numbers-in-the-united-kingdom-msc-position-paper-octob

(2) Rowland E, Trueman H. Improving healthcare student experience of clinical placements. BMJ Open Quality2024;13:e002504
(3) Anders Ericsson K. Deliberate practice and acquisition of expert performance: a general overview. Academicemergency medicine. 2008 Nov;15(11):988–94.
(4) Tyrrell E, et al. Use of an artificial intelligence driven voice recognition platform for training communication skills inundergraduate primary care. [Conference presentation] SAPC ASM 2024 Bristol.
(5) Turkelson C, Yorke AM, Keiser M, Smith L, Gilbert GE. Promoting Interprofessional Communication with VirtualSimulation and Deliberate Practice. Clinical Simulation in Nursing [Internet]. 2020 Jul 25;46:30–9. Available from: https://doi.org/10.1016/j.ecns.2020.03.008

## O25. Staging Change: The Use of Interprofessional Forum Theatre to Confront Oral Health Inequalities for Learning Disability Populations

### Format: Descriptive Work - Oral Presentations, Short Communications and E-posters

#### Topic: Culture, Wellbeing, Equity, Diversity, Inclusivity

##### Hannelie Edgar^1^, Vicky Adams^2^, Gerard McWilliams^2^, Ailish McMeel^2^, Gerry Gormley^2^

###### ^1^Northern Ireland Medical and Dental Training Agency (Belfast, Northern Ireland); ^2^Queen's University Belfast (Belfast, Northern Ireland)

*Advances in Simulation 2025,* **10(1)**:025

**Introduction: setting, background and identification of needs leading to the initiative**

**Setting**

A Forum Theatre workshop for undergraduate students from the School of Dentistry and School of Nursing and Midwifery, Queen’s University, Belfast. This will explore barriers to oral-health related care for adults with learning disabilities and how care may be improved.

**Background**

In the UK, there are approximately 1.5 million people with learning disabilities, affecting 2.16% of adults.1 These individuals face significant barriers to accessing quality healthcare, attributed to a lack of accessibility, poor staff understanding and training, a lack of interdisciplinary team working and inadequate follow-up care.2 Adults with learning disabilities often have worse oral health with increased periodontal disease and untreated decay compared with the general population.3 Forum Theatre is a form of interactive drama which has been used in healthcare education to explore themes including racial discrimination, obstetric violence and health promotion.4,5

**Objectives**

Our objectives are to:

Highlight the oral health inequalities faced by adults with learning disabilities

Encourage interdisciplinary learning and problem solving

Improve awareness of reasonable adjustments

Develop students'patient management skills

**Description of initiative and approach/methods used**

The ‘Staging Change’ simulation is a two-hour workshop for 3rd year dentistry and 2nd year learning disability nursing students, with 20 participants overall. Students watch a live-play demonstrating the difficulties a person with learning disabilities can experience when they require dental treatment. The students participate in break-out groups to explore themes raised by the scenario and suggest reasonable adjustments to improve the individual’s experience and outcomes. The play is then re-enacted with students incorporating these suggestions, and the session concludes with a structured debrief. Pre- and post-simulation questionnaires are completed alongside a focus group to draw out student feedback and experience.

**Discussion of the impact/outcome, and novelty of the initiative**

This is a novel use of Forum Theatre in undergraduate interdisciplinary education to explore health inequity for adults with learning disabilities. Findings from the workshop will be shared at the conference. We anticipate that the workshop will support the training of empathetic healthcare professionals, who make reasonable adjustments in their clinical practice to promote health equity. The use of Love/Break-up letters for participant feedback builds on previous research which encouraged use in healthcare education to promote openness.6 It is hoped that colleagues in simulation education will be inspired to consider Forum Theatre as a means of exploring themes relating to culture, wellbeing, equity, diversity and inclusivity.

**Keywords**

Dentistry, Learning Disability Nursing, Undergraduate Education

**References/Acknowledgements**

- Allerton L, Emerson E. British adults with chronic health conditions or impairments face significant barriers to accessing health services. Public health. 2012 Nov 1;126(11):920-7.
- Anders PL, Davis EL. Oral health of patients with intellectual disabilities: a systematic review. Special care in dentistry. 2010 May;30(3):110-7.
- Ward LM, Cooper SA, Hughes, McCormack L, Macpherson L, Kinnear D. Oral health of adults with intellectual disabilities: a systematic review. Journal of Intellectual Disability Research. 2019 Nov;63(11):1359-78.
- Mathonnet A, Arvis P. Training of Health Care personnel on Obstetric Violence: An Experience in Togo using the Forum Theatre Technique. Training.;1(1–2023).
- Middlewick Y, Kettle TJ, Wilson JJ. Curtains up! Using forum theatre to rehearse the art of communication in healthcare education. Nurse education in practice. 2012 May 1;12(3):139-42.
- Laughey WF, Brown ME, Liu A, Dueñas AN, Finn GM. Love and breakup letter methodology: a new research technique for medical education. Medical education. 2021 Jul;55(7):818-24.

## O26. Systems and Sayings: innovative Sim Study Days for International Medical Graduates to address unfamiliar systems and communication barriers

### Format: Descriptive Work - Oral Presentations, Short Communications and E-posters

#### Topic: Culture, Wellbeing, Equity, Diversity, Inclusivity

##### MooiSia Michelle Huang^1^, Thomas O'Neill^2^, Goran Zangana^2^

###### ^1^Royal Hospital for Children and Young People (Edinburgh, Scotland); ^2^NHS Lothian (Edinburgh, Scotland)

*Advances in Simulation 2025,* **10(1)**:026

**Introduction: setting, background and identification of needs leading to the initiative**

International medical graduates (IMGs) are diverse group who represent a significant proportion of the medical workforce, with more than half (52%) of doctors starting work in the UK in 2022 having qualified overseas(1) (an increase of 121% since 2017(1)).

Communication barriers with patients and colleagues are the most reported challenge by IMGs entering unfamiliar systems(2), exacerbated by limited access to performance feedback(2). Culture shock and disorientation are also common occurrence reported on starting work(3).

NHS Lothian’s innovative IMG Sim Study Day – which aligns with the Softer Landings, Safer Care framework(4) – aims to both overcome systems unfamiliarity and address communications barriers. The Sim Study Day combines immersive clinical and communications simulation with facilitated group study sessions, additionally augmented by an innovative AI-powered communication simulation platform to enable on-demand iterative conversation rehearsal.

**Description of initiative and approach/methods used**

The IMG Sim Study Day – designed by a faculty group of IMGs, for IMGs – consists of three main sessions: 1) hi-fidelity immersive simulation (managing the clinically unwell patient), 2) difficult conversations role-play (breaking bad news and challenging conversations), and 3) Goldfish Bowl simulation for escalating concerns and handling complaints. These simulation sessions are complimented by facilitated group discussions around bias and challenging behaviours, with the whole day incorporating a range of simulation pedagogies.

The induction is further augmented using innovative AI-powered communication simulation software (through a platform called SimConverse) to enable deliberate rehearsal of communication skills supported by iterative, comprehensive performance feedback. The platform – which has been shown to improve communication skill aquisition(5) – provides IMGs with post-study day continuation of self-regulatory learning.

**Discussion of the impact/outcome, and novelty of the initiative**

To date, 65 IMGs have participated in the Sim Study Days. Their experiences of systems unfamiliarity and communication barriers align with the national picture, and feedback suggests the Sim Study Day starts to address these areas in a constructive way.

Fostering an active support network for new IMGs entering the organisation, to cushion the impact of culture shock and disorientation, was a hidden curriculum agenda of the Sim Study Day. This was successfully reflected in feedback, with almost all participants highlighting the benefits of networking as a direct result of the session.

Access to custom-designed scenarios tailored specifically to the needs of IMGs, especially with the additional scenario accessibility via the AI-powered platform (SimConverse), has provided opportunity for skill acquisition and detailed performance feedback beyond what would otherwise be available to IMGs entering our workforce.

**Keywords**

NA

**References/Acknowledgements**

- 1. GMC (online). Workforce report 2023. The state of medical education and practice in the UK (Accessed 1 October 2024). Available from https://www.gmc-uk.org/-/media/documents/workforce-report-2023-full-report_pdf-103569478.pdf
- 2.NHS England. Workforce Race Equality Standard data reporting (2019). Available: https://www.england.nhs.uk/publications/workforce-race-equality-standard-data-reporting-2019/
- 3. Al-Haddad M, Jamieson S, Germeni E. International medical graduates'experiences before and after migration: A meta-ethnography of qualitative studies. Med Educ. 2022; 56(5): 504–515. doi:https://doi.org/10.1111/medu.14708
- 4. Trainer Resources - Softer Landing, Safer Care - Scotland Deanery. (2024). Nhs.scot. https://www.scotlanddeanery.nhs.scot/international-medical-graduates/trainer-resources-softer-landing-safer-care/
- 5. Tyrrell e, et al. Use of an artificial intelligence driven voice recognition platform for training communication skills in undergraduate primary care. (Conference presentation) SAPC ASM 2024 Bristol.

## O27. The Applicability of Existing Frameworks for Measuring Behavioural Skills in Pharmacy Practice

### Format: Research Studies - Oral Presentations, Short Communications and E-posters

#### Topic: Interprofessional/Team Education and Training

##### Julia Weber^1^, Samuel S. Allemann^1^, Kirsty Freeman^2^, Rhonda Clifford^2^, Liza Seubert^2^

###### ^1^University of Basel (Basel, Switzerland); ^2^The University of Western Australia (Perth, Australia)

*Advances in Simulation 2025,* **10(1)**:027

**Introduction: context and hypothesis/aims**

The evolving role of pharmacists requires the acquisition of behavioural skills for person-centred care (1). These skills encompass cognitive and interpersonal abilities vital for enhancing performance in complex healthcare systems, reducing errors, and ensuring safe practice. Core behavioural skills include communication, leadership, teamwork, situational awareness, decision-making, resource management, and professionalism (2). Simulation-based learning plays a pivotal role in developing these skills, providing a safe and controlled environment where learners can deepen their understanding of complex systems and human interactions (2,3). Yet, despite the increasing importance of behavioural skills, evidence on measuring them within pharmacy practice remains limited. Although existing frameworks, such as the Non-Technical Skills for Surgeons (4) and Anaesthetists (5), have been widely utilised in clinical settings, their applicability to pharmacy practice has not been established. This research aims to assess the applicability of these existing behavioural skills frameworks within the context of pharmacy practice.

**Methods and results: description of the methods used/study design/data collection. Presentation of the results addressing the study hypothesis/aims**

Second-year master's students in pharmacy will participate in summative Objective Structured Clinical Examinations designed to engage with simulated participants. Students’ behavioural skills performances will be measured by analysing the recorded Objective Structured Clinical Examinations using existing frameworks such as Non-Technical Skills for Surgeons and Anaesthetists. Applying the existing frameworks will initially facilitate the identification of gaps in behavioural skills required for pharmacy practice. The gap analysis will provide a systematic baseline, that highlights discrepancies between the current frameworks and the specific behavioural skills necessary for pharmacy practice.

**Discussion of the impact/outcome, and novelty of the Research**

The findings will offer a strong rationale for adapting the existing frameworks to better align with the competencies required in pharmacy settings. By establishing a tailored framework for measuring behavioural skills in pharmacy practice, this research aims to enhance pharmacy education via simulation-based learning and ultimately improve person-centred care. Future work will focus on implementing the adapted frameworks in educational pharmacy settings including digital simulation.

**Keywords**

Gap Analysis, Behavioural Skills, Patient-Centred Care, Pharmacy Practice, Simulation-Based Learning

**References/Acknowledgements**

1. Diec S, Patel PH, Samuel NG, Hernandez-Munoz JJ. Student perceptions of non-technical skills development duringadvanced pharmacy practice experiences. Curr Pharm Teach Learn. 2021;13(11):1510–6.
2. Lioce L, Lopreiato J, Downing D, Chang TP, Robertson JM, Anderson M, et al. Healthcare Simulation Dictionary–Second Edition. In: Rockville, MD: Agency for Healthcare Research and Quality; 2020.
3. Nestel D, Kelly M. An introduction to healthcare simulation. In: Debra Nestel, Michelle Kelly, Brian Jolly, MarcusWatson, editors. Healthcare simulation education: Evidence, theory and practice, Wiley; 2017.
4. Flin R, Yule S, Paterson-Brown S, Rowley D, Maran N. The non-technical skills for surgeons (NOTSS) systemhandbook v1. 2. 2006 [cited 2024 Oct 17]; Available from: http://dme.childrenshospital.org/wp-content/uploads/2019/03/NOTSS-handbook-v1.22.pdf
5. Flin R, Patey R, Glavin R, Maran N. Anaesthetists’ non-technical skills. BJA: British Journal of Anaesthesia.2010;105(1):38–44. https://doi.org/10.1093/bja/aeq134

## O28. The Role of Simulation in Expressing Empathic Behavior in Medical Students

### Format: Research Studies - Oral Presentations, Short Communications and E-posters

#### Topic: Curriculum Development and Assessment

##### Silvia Oldani, Claudia Ebm, Valeriano Vinci, Riccardo Sarti, Pamela Panico

###### Humanitas University (Milan, Italy)

*Advances in Simulation 2025,* **10(1)**:028

**Introduction: context and hypothesis/aims**

Compassion is a cornerstone of effective medical practice, significantly impacting patient outcomes by fostering trust and adherence to treatment plans. While some aspects of compassion are inherent, medical education plays a crucial role in refining and enhancing these behaviors. Recently, there has been a growing emphasis on incorporating compassion training into medical curricula, yet questions remain about the most effective methods for cultivating these skills. This study focuses on evaluating the role of simulation-based training in promoting empathic behavior among medical students, comparing it to traditional clinical clerkships.

**Methods and results: description of the methods used/study design/data collection. Presentation of the results addressing the study hypothesis/aims**

This retrospective longitudinal study examined 133 medical students at Humanitas University, Milan, Italy, from 2021 to 2024. The university’s curriculum includes both hospital-based clerkships and simulation-based training designed to develop professional competencies, including compassion. Students'empathic behaviors were assessed quarterly using a standardized compassion scorecard, with additional evaluations conducted through a learning management system and objective structured clinical examinations (OSCEs). The study aimed to determine how effectively simulation-based training fosters the expression of empathy compared to real-world clinical experiences.

At the outset, no significant differences were found in compassion scores between students in simulation-based settings and those in clinical clerkships (simulation: 3.30 ± 0.69, clerkship: 3.25 ± 0.73, p = 0.45). Over time, students in traditional clerkships demonstrated a steady increase in compassion scores, reflecting the deepening of empathic behavior through ongoing patient interactions (final clerkship score: 3.54 ± 0.78, p = 0.023). In contrast, while students in simulation-based training initially showed slight improvements, their compassion scores peaked in Year 4 and then declined, returning to near initial levels by the final evaluation (final simulation score: 3.23 ± 1.18, change: −0.07, 95% CI [−0.24, 0.11]).

**Discussion of the impact/outcome, and novelty of the Research**

The findings suggest that while simulation-based training is a valuable tool for introducing and practicing empathic behavior in a controlled setting, it is less effective in sustaining these behaviors over time compared to real-world clinical experiences. The lack of continuous, genuine patient interaction in simulations may limit students’ ability to fully develop and maintain compassion. Therefore, to foster lasting empathic behaviors, medical curricula should emphasize the integration of simulation with ongoing, real-world clinical exposure, ensuring that students can translate learned skills into compassionate practice.

**Keywords**

Empathy, Soft-skill training,

**References/Acknowledgements**

- Malenfant S, Jaggi P, Hayden KA, Sinclair S. Compassion in healthcare: an updated scoping review of the literature. BMC Palliat Care. 2022;21(1).
- Sinclair S, Kondejewski J, Jaggi P, Roze des Ordons AL, Kassam A, Hayden KA, et al. What works for whom in compassion training programs offered to practicing healthcare providers: a realist review. Vol. 21, BMC Medical Education. 2021

**Fig. 1 (O28):**
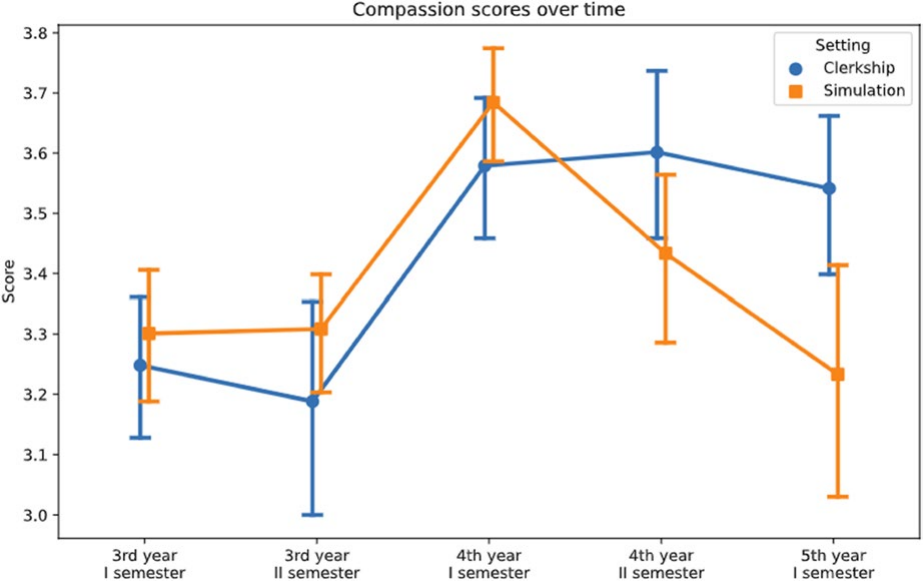
checklist for reporting of studies of reliability and agreement

## O29. The Simulation Solution: Empowering Hospital Doctors To Care For Older Adults

### Format: Research Studies - Oral Presentations, Short Communications and E-posters

#### Topic: Research Methodolgy

##### Emily Buckley^1,2^, John Cooke^2^, Deirdre Bennett^1^, Colm O'Tuathaigh^1^, Aileen Barrett^3^

###### ^1^Medical Education Unit, University College Cork (Ireland); ^2^Waterford Integrated Care for Older People (Ireland); ^3^Irish College of General Practitioners (Ireland)

*Advances in Simulation 2025,* **10(1)**:029

**Introduction: context and hypothesis/aims**

The rapidly increasing older adult population necessitates training in gerontological competencies for all hospital doctors regardless of specialty. Experiential learning and in particular, simulation-based education (SBE) has been proposed as a potential effective educational intervention to achieve this. However, research in gerontological simulation is limited and focuses on evaluation of confidence and knowledge outcomes rather than tangible changes in clinical behaviour. This study aims to explore if SBE fosters transformative learning (TL) and influences clinical behaviour in hospital doctors caring for older adults. Specifically, we explored if and how SBE can facilitate the acquisition of a specific set of gerontological competencies.

**Methods and results: description of the methods used/study design/data collection. Presentation of the results addressing the study hypothesis/aims**

Incorporating the instructional design approach- the ADDIE model (Analysis, Design, Development, Implementation, Evaluation)1 and Mesirow’s transformative learning theory2, we developed a simulation scenario focusing on the management of an older adult with delirium and Parkinson’s disease. A scoping review and national consensus mapping study were conducted to determine learning needs of hospital doctors. Doctors from all specialties within a single hospital site were invited to participate in the simulation scenario, debrief. The learners in each scenario were invited to participate in an individual semi-structured interview two to four weeks post the simulation scenario. The scenario was facilitated utilising minimal resources and two embedded simulation participants from the gerontological simulation faculty. Evaluation was carried out via an audio-recorded debrief and semi-structured interview. Debrief and interview questions were guided by the ten phases of Mesirow’s transformative learning theory. Transcripts were analysed using thematic analysis.

Nine simulation scenarios and debriefs were followed by nine individual semi-structured interviews. Participants included hospital doctors from diverse specialties including internal medicine, surgery and obstetrics and gynaecology. Participants experienced phases one to ten of TL to varying extents. Four overarching themes continuously arose contributing to our understanding of TL through SBE: 1.) ‘Creating a realistic challenge’; 2.) ‘SBE as a catalyst for reflection’; 3.) ’Looking to the future’ and 4.) Gerontological simulation: a paradigm shift’.

**Discussion of the impact/outcome, and novelty of the Research**

Simulation-based education can promote transformative learning of gerontological competencies pertaining to delirium and Parkinson’s disease for hospital doctors to varying extents. This is in keeping with similar studies that have adopted transformative learning in SBE for healthcare education 3,4. This can be achieved using minimal resources. Future research should focus on exploring how SBE can foster the transformative learning of broader gerontological competencies for this cohort. These findings could subsequently guide the development of dedicated simulation curricula for postgraduate medical training for all hospital specialties.

**Keywords**

Geriatric medicine, simulation-based education, transformative learning theory

**References/Acknowledgements**

- (1) Molenda M, Reigeluth CM, Nelson LM. Instructional design. In: Nadel L, editor. Encyclopedia of Cognitive Science: Nature Publishing Group; 2003.
- (2)Mezirow J. Transformative Dimensions of Adult Learning. San Francisco, CA: Jossey-Bass; 1991
- (3)Tallentire VR, Kerins J, McColgan-Smith S, Power A, Stewart F, Mardon J. Exploring transformative learning for trainee pharmacists through interprofessional simulation: a constructivist interview study. Advances in Simulation. 2021;6(1):31.
- (4)Kerins J, Smith SE, Phillips EC, Clarke B, Hamilton AL, Tallentire VR. Exploring transformative learning when developing medical students'non-technical skills. Medical Education. 2020;54(3):264-74.

## O30. Existe diferencia en la evaluación de la experiencia de simulación según el grado de participación?

### Format: Research Studies - Oral Presentations, Short Communications and E-posters

#### Topic: Quality assurance, Faculty development and Program evaluation

##### Carolina Astoul Bonorino, Dolores Latugaye

###### Universidad Austral (Buenos Aires, Aregentina)

*Advances in Simulation 2025,* **10(1)**:030

**Introduction: context and hypothesis/aims**

El estándar de Evaluación del Aprendizaje y Desempeño de la INACSL(1), propone la evaluación de toda experiencia basada en simulación. La participación en la simulación puede ser activa (rol profesional) o pasiva (observador). Sin embargo, todos participan de la reflexión o debriefing. Según la evidencia (2), la participación activa en las experiencias basadas en simulación pueden mejorar los resultados de aprendizaje, aunque el rol observador puede ser positivo en algunas circunstancias. Por lo expuesto, el propósito de este estudio es comparar si existe diferencia en la evaluación de la experiencia de simulación en estudiantes de enfermería en nuestro medio.

**Methods and results: description of the methods used/study design/data collection. Presentation of the results addressing the study hypothesis/aims**

Estudio observacional, analítico, realizado a partir de una base de datos. Se incluyeron todos los registros de estudiantes de grado de Enfermería de las actividades de simulación clínica del 2023 y 2024. Los escenarios se realizaron con un máximo de 12 estudiantes, que participaron de manera activa: en su rol profesional; o pasiva: observador o participante simulado. Se analizaron el aprendizaje y la confianza de la herramienta SET-M; y la percepción de aprendizaje en el debriefing (DBF) y la evaluación global del escenario. Tamaño muestral estimado: n=328 (nivel de confianza: 99%; poder: 80%; diferencia de proporciones: 10%).

Se obtuvieron 587 registros, 337 con participación activa y 250 con participación pasiva.

**Resultados**:

El 82% de los participantes activos (PA) refirió un alto nivel de aprendizaje y el 77% de los participantes pasivos (PP) refirió ese nivel de aprendizaje (p=0.433).

El 81%% de los PA refirió un nivel de confianza alto y un 78% del grupo PP (p=0.688).

En relación a si el DBF favoreció su aprendizaje, el grupo PA asintió en un 97% y el grupo PP un 96%% (P=0.174).

La media de evaluación global del escenario fue de 96% en el grupo PA y de 95% en el grupo PP (p=0.612).

**Discussion of the impact/outcome, and novelty of the Research**

Si bien la evidencia sugiere que la participación activa puede mejorar los resultados de aprendizaje, en este estudio se observa que no hay diferencia estadísticamente significativa en cuanto a la percepción de aprendizaje y confianza de los participantes, así como tampoco la evaluación global de los escenarios. Esto nos permite seguir trabajando con grupos de entre 10 y 12 estudiantes, donde algunos estudiantes participan de manera activa y otros de manera pasiva.

**Keywords**

Participación; Simulación clínica.

**References/Acknowledgements**

- I. NACSL Standards Committee (2016, December). INACSL standards of best practice: SimulationSM Participant evaluation. Clin Simul Nurs. 2016 Dec 1;12:S26–9. https://doi.org/10.1016/j.ecns.2016.09.009
- 2. Delisle M, Ward MAR, Pradarelli JC, Panda N, Howard JD, Hannenberg AA. Comparing the Learning Effectiveness of Healthcare Simulation in the Observer Versus Active Role: Systematic Review and Meta-Analysis. Vol. 14, Simulation in Healthcare. Lippincott Williams and Wilkins; 2019. p. 318–32. https://doi.org/10.1097/SIH.0000000000000377

## O31. “The power of touch” Medical students’ experiences on interaction skills in and through drama-based approaches

### Format: Research Studies - Oral Presentations, Short Communications and E-posters

#### Topic: Culture, Wellbeing, Equity, Diversity, Inclusivity

##### Minna-Maria Mattila

###### University of Helsinki (Helsinki, Finland)

Advances in Simulation 2025, **10(1)**:031

**Introduction: context and hypothesis/aims**

Medicine is an embodied practice. There is not only a cognitive level, people process, collect, create, and transform information through the body. People come to know themselves, others, the world by using bodily sensations, gestures, movements, and interactions [1–4].

In healthcare professionals are required to work in teams. Attentiveness is essential in teamwork interaction, team members need to work together, communicate, and cooperate in a safe manner and remain alert in patient situations. Also, healthcare professionals should attentively listen to what the patient is saying and experiencing, focus on being open [5,6].

This qualitative master’s thesis study aimed to find out a small group of medical students’ (n=7) perceptions and experiences on strengthening interaction skills in and through drama-based approaches in a facilitator-led setting. The study was centered around an embodiment standpoint, through which participants’ descriptions were analysed. The research question was: In what ways can drama-based approaches serve in strengthening medical students’ interaction skills?

**Methods and results: description of the methods used/study design/data collection. Presentation of the results addressing the study hypothesis/aims**

The workshop exercises focused on adding presence and attentiveness in interaction and facilitating reflection in action.

Volunteer students attended the one-time-workshop. The data was collected using post-workshop questionnaires (immediate and afterwards) with open questions and analysed by using thematic analysis.

Five common themes from the students’ answers emerged: 1) Interaction skills are embodied, 2) Increased awareness of the other person and the sense of belonging, 3) Means to promote wellbeing, 4) Sense of embodied cognition, and 5) Drama-based workshop well-accepted.

The findings indicated that the students were experiencing the contacts with the patients in a different way than before the workshop. The participants aimed to use explicit ways how to communicate verbally and being present in interaction situations. Also, they paid attention to their own wellbeing by consciously calming their minds. The workshop seemed to facilitate students’ understanding of the embodiment and its importance in interaction [7].

**Discussion of the impact/outcome, and novelty of the Research**

Drama-based workshop was well-accepted by participant students. Experiencing and reflecting through active exercises and joint discussions seemed to facilitate becoming aware of means how to strengthen interaction. For example, the students became aware of touch as a way to listening, being present, showing empathy and sharing emotions. These experiences might evoke them to think about not only the body but lived experience of the person when working with patients [8, 9].

The findings encourage educational interventions for developing awareness and mind-body connection via embodied approaches. This kind of workshop could serve as warm-up for simulation training.

**Keywords**

Attentiveness, drama-based approaches, embodied cognition, embodiment, interaction skills, medical humanities, medical students, N.A.

**References/Acknowledgements**

- Merleau-Ponty, M. (2012). Filosofisia kirjoituksia. Toimittaneet ja suomentaneet Miika Luoto ja Tarja Roinila. Kustannusosakeyhtiö Nemo. Helsinki.
- Shapiro, L. (2019). Embodied Cognition. Second edition. Routledge, U.K.
- Sutela, K., Kivijärvi, S. & Anttila, E. (2021). Moving encounters: Embodied pedagogical interaction in music anddance educators’ expanding professionalism. 10.4324/9781003108337–8.
- Loue S. (2022). Teaching and Practicing Humanism and Empathy through Embodied Engagement. Medicina (Kaunas, Lithuania), 58(3), 330. https://doi.org/10.3390/medicina58030330
- Schmutz, Jan B. & Meier, Laurenz L. & Manser, Tanja 2019. How effective is teamwork really? The relationshipbetween teamwork and performance in healthcare teams: a systematic review and meta-analysis. BMJ Open. https://bmjopen.bmj.com/content/9/9/e028280.
- Adamson K, Loomis C, Cadell S, Verweel LC. (2018), Interprofessional empathy: A four-stage model for a newunderstanding of teamwork. J Interprof Care. 2018 Nov;32(6):752–761. https://doi.org/10.1080/13561820.2018.1511523. Epub 2018 Aug 30. PMID: 30,160,548.
- Mattila, Minna-Maria. (2023). To be seen and heard: Medical students’ experiences on interaction skills in andthrough drama-based approaches. Metropolia University of Applied Sciences, Master’s Thesis. https://urn.fi/URN:NBN:fi:amk-2023120133443
- Kelly, M., Ellaway, R., Scherpbier, A., King, N., & Dornan, T. (2019). Body pedagogics: embodied learning for thehealth professions. Medical education, 53(10), 967–977. https://doi.org/10.1111/medu.13916
- Kelly, M., Svrcek, C., King, N., Scherpbier, A., & Dornan, T. (2020). Embodying empathy: A phenomenological studyof physician touch. Medical education, 54(5), 400–407. https://doi.org/10.1111/medu.14040

